# Human Face-Selective Cortex Does Not Distinguish between Members of a Racial Outgroup

**DOI:** 10.1523/ENEURO.0431-19.2020

**Published:** 2020-05-29

**Authors:** Niv Reggev, Kirstan Brodie, Mina Cikara, Jason P. Mitchell

**Affiliations:** 1 Department of Psychology, Harvard University, Cambridge, MA 02138; 2 Department of Psychology, Ben Gurion University of the Negev, Be’er-Sheva 84105, Israel; 3 Zlotowski Center for Neuroscience, Ben Gurion University of the Negev, Be’er-Sheva 84105, Israel

**Keywords:** cross-race, faces, FFA, fMRI, outgroup homogeneity, repetition suppression

## Abstract

People often fail to individuate members of social outgroups, a phenomenon known as the outgroup homogeneity effect. Here, we used functional magnetic resonance imaging (fMRI) repetition suppression to investigate the neural representation underlying this effect. In a preregistered study, White human perceivers (*N *=* *29) responded to pairs of faces depicting White or Black targets. In each pair, the second face depicted either the same target as the first face, a different target from the same race, or a scrambled face outline. We localized face-selective neural regions via an independent task, and demonstrated that neural activity in the fusiform face area (FFA) distinguished different faces only when targets belonged to the perceivers’ racial ingroup (White). By contrast, face-selective cortex did not discriminate between other-race individuals. Moreover, across two studies (total *N *=* *67) perceivers were slower to discriminate between different outgroup members and remembered them to a lesser extent. Together, these results suggest that the outgroup homogeneity effect arises when early-to-mid-level visual processing results in an erroneous overlap of representations of outgroup members.

## Significance Statement

Researchers have repeatedly demonstrated that perceivers struggle to distinguish between different members of a racial outgroup. Here, we show in a preregistered study that this failure arises when areas of the human brain that specifically process facial identity, most notably, the so-called “fusiform face area” (FFA), fail to detect differences between identities of members of a racial outgroup. When White perceivers viewed photographs of two different Black men, the face area of their brains responded as if the two photographs portrayed the same person. This effect was constrained to outgroup faces; the face area successfully distinguished faces of two different White individuals. Our results highlight the failure of basic representational mechanisms in processing individuals from other social groups.

## Introduction

The outgroup homogeneity effect (sometimes called the cross-race effect) describes the difficulty people often experience when trying to identify members of a racial outgroup. Although race is not a valid biological taxonomy, individuals in our society define separate human races by sociocultural experiences and use this social taxonomy as a basis for numerous social and cognitive processes (Wagner et al., 2017). For example, White perceivers typically remember faces of White targets better than faces of Black targets, and are more likely to say that a new, unfamiliar Black face is the same as one they have seen previously (Malpass and Kravitz, 1969; Meissner and Brigham, 2001). Perceivers also identify own-race faces faster and more accurately than cross-race faces (Marcon et al., 2010). This pattern of (mis)identification has demonstrable societal consequences. In an analysis of American police proceedings, White witnesses correctly identified 60% of perpetrators when a line-up comprised other White individuals but identified only 45% of Black perpetrators; more than half the time, unrelated (i.e., innocent) Black individuals were identified as perpetrators (Behrman and Davey, 2001).

Most theories of the outgroup homogeneity effect suggest that perceivers create detailed, individuated representations of ingroup members, but view outgroup members as interchangeable instances of a category (Papesh and Goldinger, 2010; Hugenberg et al., 2013; Correll et al., 2017). Consequently, scholars hypothesize that this differential representation underlies perceivers’ improved memory for members of their ingroup, as well as their heightened judgments of diversity and distinctiveness for ingroup targets (Park and Rothbart, 1982; Judd et al., 2005; Boldry et al., 2007). However, research to date has measured representations of racial ingroup and outgroup members mainly as a function of behavioral responses to ingroup and outgroup targets (e.g., reaction time differences between different targets; Papesh and Goldinger, 2010). To the best of our knowledge, no study has directly measured the target-specific representations of members of different groups, which we hypothesize underlie the previously mentioned behavioral results.

To bridge this gap, we conducted a functional magnetic resonance imaging (fMRI) study that made use of the phenomenon of repetition suppression, whereby neural responses to a repeated stimulus are reduced (or suppressed) relative to a sequence of two different stimuli (Gotts et al., 2012). For example, regions of the human brain that respond robustly to faces, such as the fusiform gyrus, will decrease their activity when participants view the same face repeatedly. By contrast, activation in this region will return to typically high levels of activation when a new face is presented (Gilaie-Dotan et al., 2010). Accordingly, this “release from suppression” can be used to measure the degree to which perceivers detect that they have viewed the faces of two different individuals. Thus, our approach extends previous neuroimaging studies that have, for the most part, characterized differences in mean levels of neural activity between social groups (Golby et al., 2001; Van Bavel et al., 2008; Mathur et al., 2012). Whereas these traditional univariate approaches can offer insights into the localization of neural activity differences in response to social groups, the release from suppression effect, by contrast, characterizes the uniqueness (or similarity) of representations of distinct Black and distinct White faces.

Here, we make use of the release-from-suppression logic to examine the representational basis of the outgroup homogeneity effect. Researchers have shown that the fusiform face area (FFA) shows repetition suppression even when participants see a single individual from different angles (Pourtois et al., 2005) or from different distances (Grill-Spector et al., 1999), suggesting that the FFA is sensitive to the identity of an individual rather than the similarity of their perceptual features. We capitalized on these characteristics to examine whether White perceivers will likewise represent two different Black individuals as more similar to each other than two White individuals. Specifically, we hypothesized that if perceivers individuate ingroup and outgroup faces equally, then we should observe similar release from suppression for two different face identities regardless of their race. However, to the extent that White perceivers are worse at “detecting a difference” between faces of different Black targets, then the FFA should show more suppression when a Black face is followed by a new, different Black face, despite the second face being different from the first (for related approaches, see Vizioli et al., 2010; Hughes et al., 2019). Importantly, only identity-sensitive regions such as the FFA should show the differential suppression for Black faces; face-specific identity-insensitive regions [e.g., occipital face area (OFA)] should show equivalent suppression for different Black and White faces.

**Figure 1.**
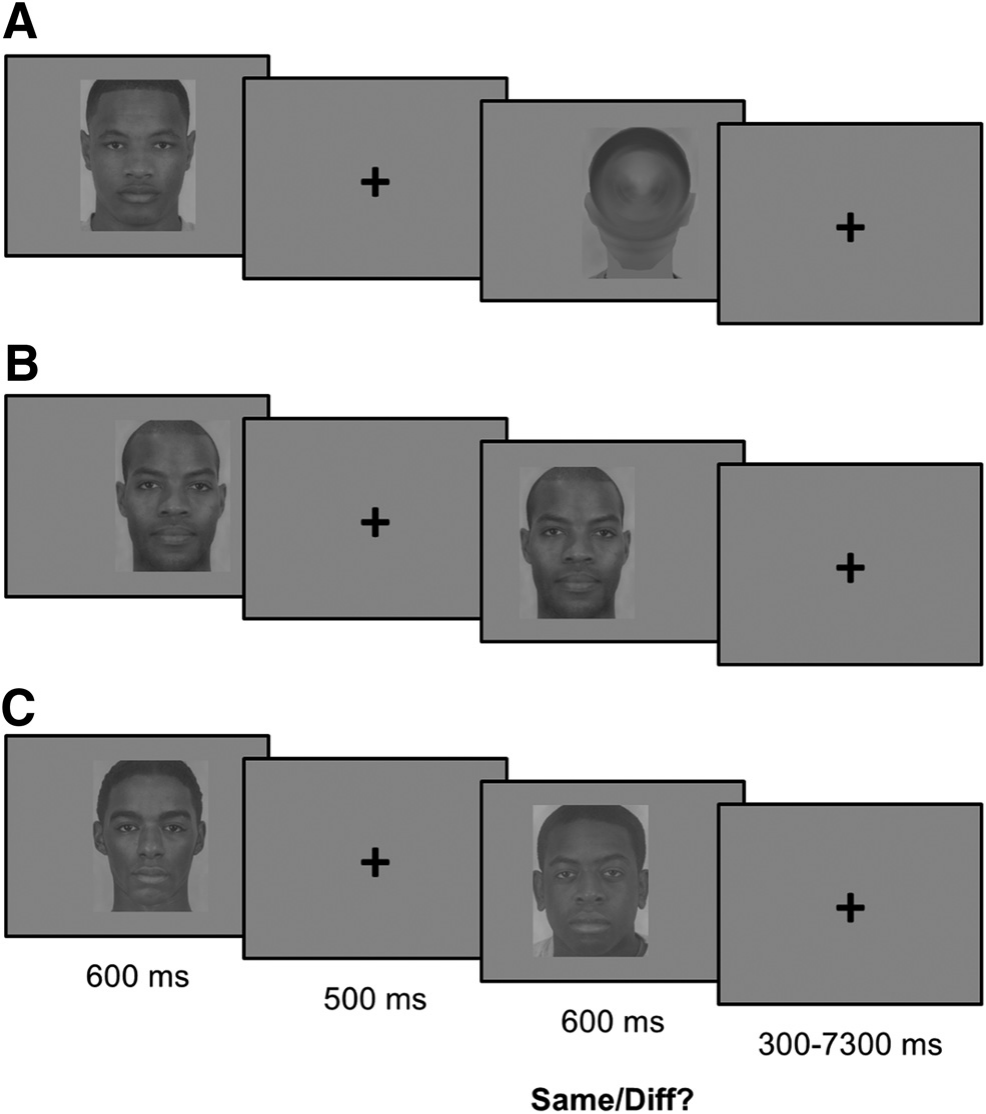
Task design. In the fMRI scanner, participants viewed face pairs from different races (Black, White) and genders (women, men) and decided if the two faces belonged to the same individual. Participants saw each face for 600 ms and responded when the second face was on screen. Faces were presented with a random horizontal jitter to prevent low-level suppression effects. The design included three face pair conditions for each combination of race and gender. Participants saw (***A***) a face followed by a scrambled visual patch in the shape of a face in the single condition (i.e., baseline trials; we included this condition only in experiment 2), (***B***) a face followed by the same face in the repeated-face condition, and (***C***) a face followed by a different face (matched in gender and race) in the different-face condition. We report the behavioral results in Figure 2 (male targets) and Extended Data Figure 1-1 (female targets).

To test these predictions, we conducted a behavioral experiment and a preregistered fMRI study using a repetition suppression paradigm. In each experiment, White participants sequentially viewed pairs of faces that varied in race (Black, White) and gender (woman, man). We matched face categories in perceptual and structural properties (see Materials and Methods). For each pair, participants indicated whether the faces were of the same or different individuals. In some trials, the two faces were identical; in an equal number of trials, the two faces depicted different individuals of the same race and gender. In addition, in one-third of trials in the fMRI experiment, the second face was replaced by a scrambled face-shaped patch; this condition allowed us to establish a difference in baseline neural processing of Black and White faces, and to directly replicate earlier studies (Golby et al., 2001; Van Bavel et al., 2011; Fig. 1). Our behavioral pilot studies indicated that participants demonstrated a reliable race effect only for male targets. Therefore, our confirmatory analyses focused on male targets; we report the results of the exploratory analyses for female targets in the supporting information. Notably, most previous studies of the other-race homogeneity effect have included only male targets; here, we provide an initial attempt to address this empirical lacuna.

10.1523/ENEURO.0431-19.2020.f1-1 Extended Data Figure 1-1 Reaction time and memory performance for female faces in experiments 1 and 2. All panels depict estimates computed by gLMMs, as detailed in Materials and Methods. ***A***, Participants in experiment 1 were faster to respond to repeated faces than to different faces (*t *=* *8.43, *p *<* *0.001). However, unlike responses for male faces, there was no evidence for an effect of race (*t *=* *0.34, *p *=* *0.74) nor for an interaction of race with type of repetition (interaction model comparison: χ_(1)_ = 1.93, *p *=* *0.16, suggesting that a model with no interaction term provides a better fit to the data). This pattern hints that Black female faces were individuated to the same extent as White female faces. When we included all trials to test a full model of the data, with the fixed effects of race, repetition, and gender, we observed a significant three-way interaction (model comparison: χ_(4)_ = 10.67, *p *=* *0.031), confirming the difference in individuation between male and female faces. ***B***, Unlike male faces, participants were not more likely to remember White female faces compared to Black female faces (OR = 1.004, Wald’s *z *=* *0.09, *p *=* *0.93). Participants were more likely to remember repeated faces compared to different faces (OR = 1.10, Wald’s *z *=* *2.20, *p *=* *0.028) with no interaction with race (OR = 1.02, Wald’s *z *=* *0.456, *p *=* *0.65). However, we did not observe a statistically significant three-way interaction effect for gender when it was inserted into the model (χ_(4)_ = 6.88, *p *=* *0.14). ***C***, Similar to responses to male faces, participants in experiment 2 responded faster to single face trials (model comparison: χ_(2)_ = 111.42, *p *<* *0.001). However, unlike response times for male faces, we did not observe a robust effect of race (*t *=* *1.97, *p *=* *0.049) nor an interaction of race and repetition (model comparison: χ_(2)_ = 1.03, *p *=* *0.597; we obtained similar results when omitting the single condition from the analysis). As in experiment 1, when we included the fixed effects of race, repetition, and gender in the model, we observed a significant triple interaction (model comparison: χ_(4)_ = 27.84, *p *<* *0.001), once again confirming the difference in processing between male and female faces. Error bars indicate SEM. Download Figure 1-1, TIF file.

## Materials and Methods

Human participants were recruited from the local community using the Harvard Department of Psychology Study Pool website. All participants provided their informed consent in a manner approved by the Committee on the Use of Human Subjects in Research at Harvard University.

### Experiment 1

Thirty-eight self-identified White participants completed the experiment (22 female, 16 male; mean age: 21.00, SD: 2.71, range: 16–29). We excluded one additional participant who failed to respond on time to >20% of the trials in the face identification task. We collected the data on a rolling basis (multiple slots per day) with a target sample size of 32 participants. We identified this sample size to be sufficient to achieve a power of 0.8 to detect a hypothesized meaningful effect size estimate (Cohen’s *d *=* *0.3, approximately equivalent to η^2^_p_ = 0.03; Cohen, 1988, pp 276–281) for the interaction contrast of race (Black/White) by condition (repeated/different). Participants completed the experiment for course credit or financial compensation (US$10).

### Experiment 2

Based on an identical power calculation, we aimed to collect analyzable data from 32 participants. To achieve this goal, we collected data from 38 self-identified White participants. In line with our preregistered exclusion criteria, we excluded six participants before data inspection due to lack of response to >20% of the trials in the main task. In the main text, we report the results from 29 participants (15 female, 13 male, one non-binary; mean age: 21.97, SD: 3.02, range: 18–28) because we excluded three additional participants whose reaction time or accuracy data were >2 SDs beyond the sample mean (we report analysis with the full sample in the supporting information—the results do not change). All 32 participants provided adequate data in terms of signal quality, as measured by a slice signal-to-noise ratio higher than 150 and by having no more than three discrete movements larger than 0.5 mm. All participants were healthy, right-handed, native English speakers with normal or corrected-to-normal vision, and no history of neurologic or psychiatric conditions. Participants completed the experiment for course credit or financial compensation (US$50).

### Materials

We obtained the face images for this study from the Chicago Face Database (CFD; Ma et al., 2015). We excluded faces that were identified as belonging to their respective racial group by fewer than 60% of the CFD independent raters, and faces that were identified as belonging to their racial group by between 60% and 75% of CFD raters were reviewed by two additional independent raters (N. R. and K. B.). Of these faces, 12 were excluded for having atypical features for their respective racial group, determined by interrater agreement between the additional raters. Lastly, all faces that were identified by 75% or more of CFD raters as belonging to their respective racial group were individually reviewed. Of these faces, two were excluded due to unique/distinctive facial features such as scars, and three were excluded due to noticeable artifacts in the image quality. This review process resulted in a final total of 94 Black female faces, 76 Black male faces, 77 White female faces, and 81 White male faces. This allowed us to select a total of 76 unique faces from each category for each participant (we used only 72 faces in experiment 1 as it required fewer stimuli; see task description below). We converted all images to greyscale, matched them on luminance (separately for foreground and background) using the SHINE toolbox (Willenbockel et al., 2010) for MATLAB (MathWorks), cropped them to 1246 by 946 pixels, matched them on spatial frequency using the SHINE toolbox, and finally, resized all photographs to 199 by 262 pixels. All face stimuli were presented in a rectangular box that included hair outline (Fig. 1).

To confirm that differences among the different categories were not confounded with image similarity, we measured the similarity between images by calculating the Structural Similarity Index (SSIM; Wang et al., 2004). This measure was computed after the experiment was completed. We computed the pairwise SSIM between all images in each category and averaged the SSIM score per image. This resulted in a vector of SSIM scores per category. Black and White men did not significantly differ in their average similarity (0.7169 vs 0.7148, respectively; *t*_(155)_ = 0.75, *p *=* *0.46). Black women, however, were less similar to each other compared with White women (0.7012 vs 0.7146, respectively; *t*_(155)_ = 5.31, *p *<* *0.001). This difference limits the potential interpretation of our exploratory analyses for female target faces (see Extended Data Fig. 3-5 and discussion below).

### Procedure

We presented all tasks and stimuli via PsychoPy v1.84.2 (Peirce, 2007) running under Mac OS X 10.7 (experiment 1) or Windows 7 (experiment 2).

### Face repetition suppression task

During the main task, we presented face images to participants in same-race, same-gender pairs. Experiment 1 included two conditions. In the repeated-face condition, the second face was identical to the first face. In the different-face condition, the second face was different from the first face. Experiment 2 included both the repeated-face condition and the different-face condition, as well as an additional single-face condition, where a single face was followed by a scrambled visual patch in the shape of a face; these pairs were used to establish a baseline. In experiment 1, participants saw 24 face pairs per condition for each race (12 pairs for each gender). Experiment 2 included 38 face pairs per condition for each race (19 for each gender).

Each trial in the face task began with a face presented for 600 ms, followed by a fixation crosshair presented in the center of the screen for 500 ms. Then, a second face, along with a response prompt, was presented for 600 ms. Lastly, a final fixation crosshair was presented for 300 ms, for a total duration of 2 s per trial. A jittered intertrial interval (range: 0–7, mean = 0.55, SD = 1.03) then followed. Participants used two fingers of their left hand to indicate, for each pair of faces, whether the second face was the same as or different from the first. Participants provided their response while the second face and response prompt were being presented on screen. The locations of both the first and second faces on the screen included a randomized horizontal offset (within a predetermined range) to minimize the interference of visual after-effects. For each participant, no single face image was used in more than one pair. The specific faces assigned to each of the conditions, as well as their pairings, were randomized between participants.

Before beginning the main task, participants practiced the task to become acquainted with trial structure and speed. In experiment 2, participants completed two rounds of practice before entering the fMRI scanner and then completed an additional practice round after entering the scanner before they started the main task. Face images used in the practice rounds were drawn from the face images that were excluded from the main task stimuli.

In experiment 2, trials were divided into two runs with an equal distribution of conditions between runs. To optimize estimation of the event-related fMRI response, conditions were intermixed in a pseudo-random order and separated by a variable, algorithm-based interstimulus interval consisting of a fixation crosshair. We used OptSeq2 (Dale, 1999) to generate sequences optimized for efficiency of the contrast (single > repeated) for a first-order counterbalanced event sequence. Of these sequences, we selected four sequences that contained no more than six consecutive trials of the same race. We randomly assigned (with replacement) an event sequence for each functional run to avoid spurious results attributable to differences between conditions in one specific event sequence (Mumford et al., 2014). Within condition, trials were presented in a unique random order for each participant. During the task, we measured behavioral task performance, including accuracy and response reaction time.

### Face functional dynamic localizer task

After completing the main task, participants in experiment 2 completed the dynamic localizer task (Pitcher et al., 2011) to localize brain regions associated with the processing of faces. Participants were informed about this task only on its execution. The dynamic localizer task instructed participants to respond via a button press to dynamic stimuli, short (3 s) movie clips of various categories. The stimuli were grouped into five categories: faces, objects, bodies, landscape scenes, and scrambled objects. All faces and bodies belonged to White individuals. We chose these specific stimuli as they have been previously validated in a dynamic task (Pitcher et al., 2011). To the best of our knowledge, no previous studies have validated non-White dynamic face stimuli. Each run was presented in the following structure: first, a fixation crosshair was presented for 18 s; then, movie clips were presented back-to-back with no intertrial interval. Movie clips were blocked by category such that each block contained six video clips, of ∼3 s each, all portraying the same category (e.g., block 1 contained only clips of faces, block 2 contained only clips of objects, etc.). Then, another fixation crosshair was presented for 18 s, followed by another series of different video clips organized like the first, but with the order of categories reversed. The run concluded with a final fixation crosshair presented for 18 s. Each run lasted 234 s, and there were four runs total. Participants were asked to press a button when they saw a repeated stimulus (a one-back repetition detection); for each run, there was one repetition within each category. We implemented the task by adapting code written by Visconti di Oleggio Castello (2017).

### Post-task behavioral measures

Upon completion of the face task, participants in experiment 1 completed a surprise recognition memory task. For this task, two faces were presented side-by-side on a screen, with one face having been presented previously, and one face being completely novel. Thirty-six pairs of faces per race per gender were presented (144 face pairs in total). Participants used four keys to indicate which was the previously presented face, given four choices: surely left, maybe left, maybe right, or surely right.

Participants in both experiments also completed the following questionnaires and behavioral measures: external and internal motivation to control prejudice (Plant and Devine, 1998), social dominance orientation (Ho et al., 2015), and the implicit association test (IAT; Greenwald et al., 1998) using Black and White faces as target stimuli with positive and negative categories (Nosek et al., 2007). Finally, to assess the degree of outgroup contact, participants responded to the following three items (all requiring open-ended responses, anchored at 0): “How many African-American friends do you have?”, “In a typical week, how many times do you meet with African-American friends?”, “How many of your close friends or family have African-American friends?”. Aside from the recognition memory task, all postscan behavioral measures and questionnaires were included for the purposes of exploratory analysis, as noted in the preregistration. It is our intent to conduct further studies, and to eventually aggregate the results of these measures across studies once a suitable power is attained.

### fMRI acquisition and preprocessing

We collected all images with a 3T Siemens Prisma scanner system (Siemens Medical Systems) using a 64-channel radiofrequency head coil. First, we acquired high-resolution anatomic images using a T1-weighted 3D MPRAGE sequence (TR = 2200 ms, TI = 1100 ms, acquisition matrix = 256 × 256 × 176, flip angle = 7, voxel size = 1 × 1 × 1 mm^3^). Second, we acquired a fieldmap in the same plane as the functional images to correct for inhomogeneities in the magnetic field (Cusack and Papadakis, 2002). Next, we collected whole brain functional images using a simultaneous multislice (multiband) T2*-weighted gradient echo sequence, sensitive to BOLD contrast, developed at the Center for Magnetic Resonance Research (CMRR) at the University of Minnesota (Feinberg et al., 2010; Moeller et al., 2010; Xu et al., 2013; TR = 2000 ms, TE = 30 ms, voxel size = 2 × 2 × 2 mm^3^, 75 slices auto-aligned to −25 degrees of the AC-PC line, image matrix = 104 × 104, FOV = 208 × 208 mm^2^, flip angle = 75°, GRAPPA acceleration factor = 2, multiband factor = 3, phase encoding direction = A -> P). Following a short in-scanner practice scan, the face repetition suppression task included two runs consisting of 188 volumes each and was followed by the dynamic face localizer task including four runs, 120 volumes each; all runs were complemented by two additional dummy scans and an initial period of ∼26 s dedicated to references for the GRAPPA procedure. The first three volumes from each run (i.e., in addition to dummy scans) were discarded to ensure T1 equilibrium. The last five volumes from the face repetition suppression runs always included a crosshair fixation to ensure the appropriate estimation of the hemodynamic function for the last events in each run.

We conducted rudimentary quality control using the recommendations for the quality control tool implemented at the scanner facility. We used SPM12 version 6225 (Wellcome Department of Cognitive Neurology) on a 2015b MATLAB platform (MathWorks) to process and analyze the fMRI data. We corrected functional data for differences in acquisition time between slices, corrected for inhomogeneities in the magnetic field using the fieldmap (Cusack and Papadakis, 2002), realigned to the first image to correct for head movement using a second degree B-spline interpolation, unwarped to account for residual movement related variance using a fourth degree B-spline interpolation, and co-registered with each participant’s anatomic data. Then, we transformed the functional data into a standard anatomic space (2-mm isotropic voxels) based on the ICBM152 brain template (Montreal Neurologic Institute). We then spatially smoothed (5-mm full-width at half-maximum) normalized data using a Gaussian Kernel.

### Statistical analysis

#### Statistical modeling, behavioral data

We analyzed reaction time and accuracy data with mixed models as implemented in the lme4 package version 1.1-14 (Bates et al., 2014) for R version 3.4.2 (R Core Team, 2017). To avoid transformation of raw reaction time data, we used generalized linear mixed models (gLMMs) with the inverse Gaussian identity link (Lo and Andrews, 2015). Memory performance in experiment 1 was analyzed using logit gLMMs with the binomial link (Jaeger, 2008). We included random effects for the intercepts for participants in all analyses. We added random intercepts for faces and by-participant random slopes for the fixed effect of race if this addition did not result in a convergence failure. Trials that elicited no response (<1.5% of all trials; no difference between conditions) were excluded from reaction time analyses.

#### Neuroimaging data

We performed statistical analyses using the general linear model (GLM) that included boxcar functions of variable duration determined per trial by reaction time to target faces (i.e., variable epochs). We chose this analysis approach to control for effects of reaction time on the neural response (Grinband et al., 2008). We set the onset of the boxcar function to the onset of prime face presentation on each trial. We deviated from the preregistered protocol by modeling single-face trials as a boxcar function with a fixed duration of 600 ms (the duration of presentation for the first face, rather than reaction time to target face) to capture true baseline activity for a single face. The reported results replicated when we conducted the analyses without this deviation (Extended Data Fig. 3-3).

The model included six conditions per gender (two races by three conditions: repeated faces, different faces, single face). We modeled trials that elicited no response in a separate regressor, and all regressors of interest were convolved with a canonical hemodynamic response function and its temporal derivative. The final first-level GLM was high-pass filtered at 128 s and included nuisance regressors specifying the six motion parameters calculated during the motion correction procedures, their temporal derivative, and a session mean per run. Preregistered validation analyses were conducted with an additional model that included an additional separate regressor for trials in which participants erred (Extended Data Fig. 3-2).

#### Regions of interest (ROIs)

We defined ROIs independently from the task localizer data by the group-constrained subject-specific method (Julian et al., 2012) as implemented in the spm_ss toolbox (Nieto-Castañón and Fedorenko, 2012). Briefly, this method was designed to discover regions that are systematically activated across participants and to define the borders around and between each of these regions. This method identifies key “parcels” within which most participants show activation for the contrast of interest. The selection of functional ROIs for individual participants is then accomplished by intersecting each individual participant’s localizer activation map with each of the parcels, thus defining functional ROIs in each individual participant in a fully algorithmic fashion. We applied this method to generate ROIs that responded to faces over all other categories (the face vs other contrasts; for the full results of this procedure, see Extended Data Fig. 3-6). We then extracted average parameter estimates across voxels from each participant-specific functional ROI using in-house scripts. We analyzed the data using a within-participant 2 (race) by 3 (condition) ANOVA as implemented by afex package (Singmann et al., 2018) for R, version 0.22-1, and plotted the results using the package ggstatsplot (Patil, 2018), version 0.2.0, and the package dabestr (Ho et al., 2019), version 0.2.2.

#### Statistical inference

For the main analysis of interest (hypotheses 5 and 6 in the preregistration), we focused on the race by condition interaction, with specific focus on two separate interaction contrasts. One interaction contrast tested activation differences between the single-face condition and the repeated-face condition as a function of race, and another interaction contrast tested activation differences between the single-face condition and the different-face condition as a function of race. Follow-up simple effects models (one model per condition) tested differences between responses to repeated and different faces separately for Black and White faces. To demonstrate no difference between conditions, we performed an equivalence test using the equivalence package for R, version 0.7.2 (Robinson, 2016).

#### Open practices

Experiment 1 was not formally preregistered. All data collection procedures and analytic choices for experiment 2 were preregistered on the Open Science Framework (OSF; https://osf.io/cw4dj/). We explicitly report any deviations from preregistration in the manuscript and Extended Data. All de-identified data and code are freely available on the OSF (https://osf.io/6z5cj/).

## Results and Discussion

### Experiment 1

**Figure 2.**
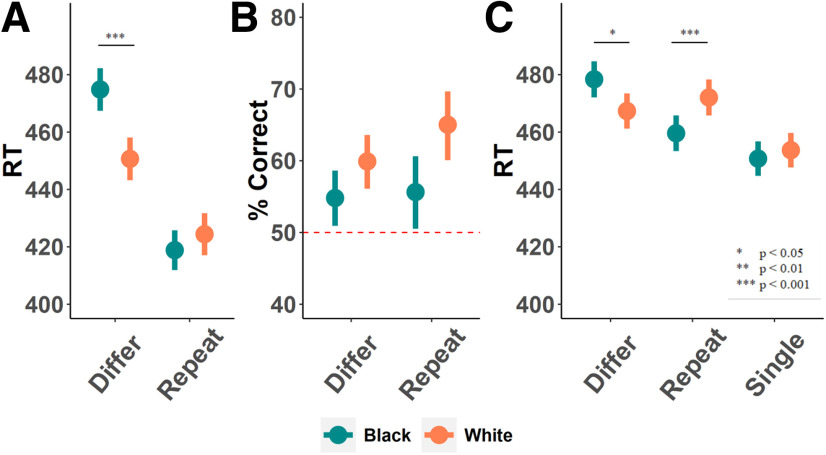
Reaction time and memory performance for male faces in experiments 1 and 2. All panels depict estimates computed by gLMMs, as detailed in Materials and Methods. ***A***, In the face repetition task in experiment 1, participants responded faster to repeated faces (*t *=* *11.33, *p *<* *0.001). This effect was qualified by an interaction with race, such that participants responded faster to different White individuals compared with different Black individuals (for inferential statistics, see main text). ***B***, In a two-alternative forced-choice test, participants in experiment 1 indicated their memory by selecting the individual they thought was presented in the face repetition task. All trials included one previously presented individual (“old”) and one never-before-seen individual (“new”). Participants remembered old White targets better than old Black targets (OR = 1.16, Wald’s *z *=* *3.005, *p *=* *0.003). We did not observe a significant effect of type of repetition on memory (OR = 1.07, Wald’s *z *=* *1.48, *p *=* *0.14) or of the interaction of repetition with race (OR = 1.05, Wald’s *z *=* *1.09, *p *=* *0.28). ***C***, Experiment 2 included an additional condition, single faces, to facilitate comparison of repetition suppression to baseline neural activity for each race. Race did not affect reaction time in the single-face condition (*t *=* *1.04, *p *=* *0.300). When analyzing only face-pair trials (omitting the single face trials), participants responded faster to repeated faces (*t *=* *2.46, *p *=* *0.014), an effect that was qualified by an interaction, replicating the results of experiment 1 (interaction model comparison: χ_(1)_ = 15.095, *p *<* *0.001): participants were slower to respond to different Black faces compared with different White faces (*t *=* *2.48, *p *=* *0.013). Unexpectedly, participants in experiment 2 were also slower to respond to repeated White faces compared with repeated Black faces (*t *=* *3.29, *p *<* *0.001). Note that unlike experiment 1, the correct response key in experiment 2 was imbalanced between the conditions; we assigned the same key to single and different conditions to simplify the task, hence assigning the same correct response key to two thirds of the trials. This design choice probably slowed the responses to repeated trials, as participants had to use an infrequent key to respond correctly to these trials. This, in turn, might have made responses to repeated targets more difficult, a difficulty that manifested particularly strongly for White targets. Error bars indicate standard error of the mean.

In experiment 1, we used a combination of memory and reaction time measures to validate our paradigm. Thirty-eight White participants first viewed 96 repeated or different face pairs (24 per gender, per race). Subsequently, these participants completed a surprise memory task in which they saw pairs of faces, with one previously seen face and one new face in each pair, and indicated which face they had previously viewed. Because pilot testing demonstrated that participants show the effect only for male targets, we report analyses limited to these faces (for analysis of behavioral responses to female faces, see Extended Data Fig. 1-1). In line with previous studies, participants were more likely to accurately remember faces of White men (61.6% correct) than faces of Black men [55.1% correct; odds ratio (OR) = 1.16, Wald’s *z *=* *3.005, *p *=* *0.003; for full results; Fig. 2*B*]. In addition, in the face repetition task, participants were quicker to identify different White faces as different (mean ± SE: 451 ± 7 ms) than different Black faces (475 ± 7 ms; *t *=* *3.664, *p *<* *0.001), but they identified Black and White repeated faces as identical equally fast (419 ± 7 vs 424 ± 7 ms for Black and White faces, respectively; *t *=* *0.568, *p *>* *0.5; interaction model comparison: χ_(1)_ = 16.89, *p *<* *0.001; Fig. 2*A*). In other words, participants showed reduced memory and slower responses for male outgroup faces, particularly when they viewed two different individuals from each group. This suggests that although perceivers can successfully process a target from an outgroup if they see it repeatedly, they treat different outgroup faces as more homogenous to one another relative to different ingroup faces.

### Experiment 2

**Figure 3.**
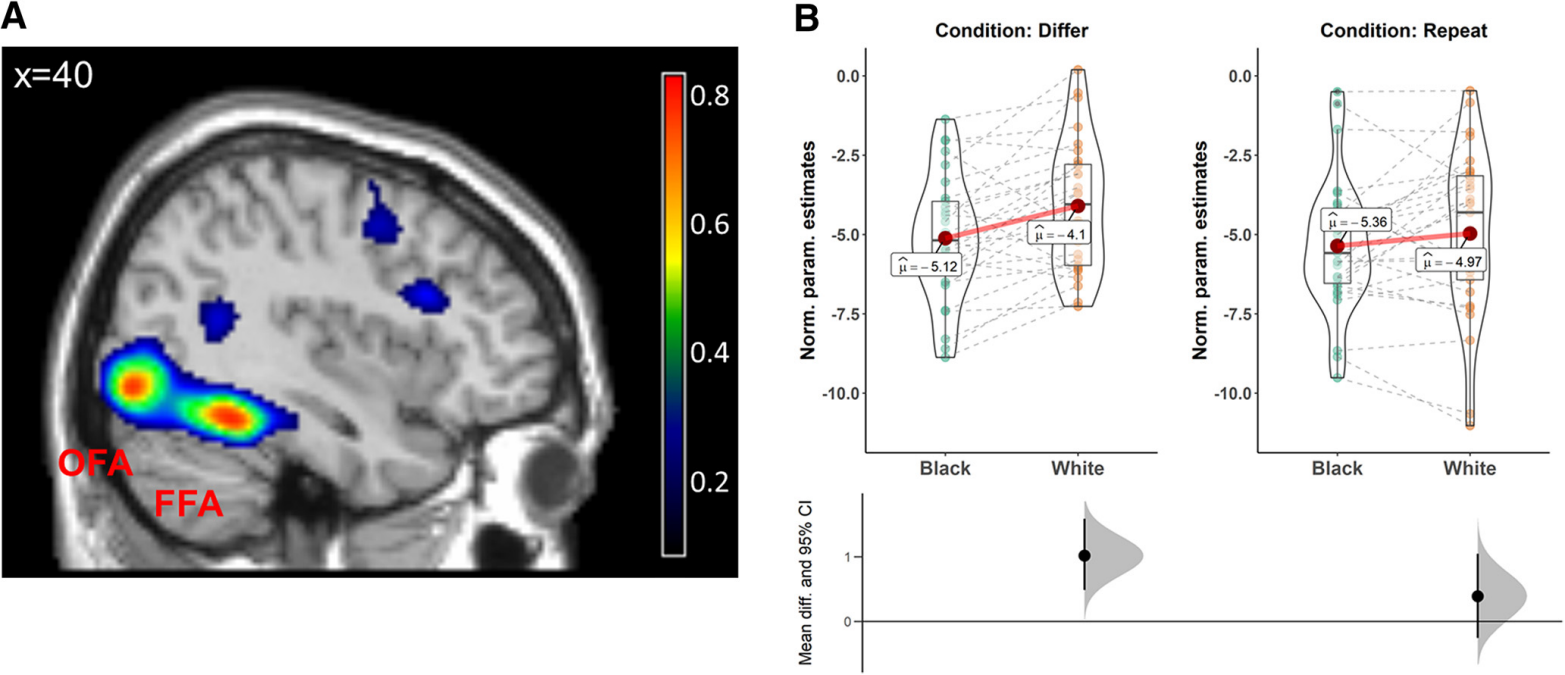
Results of the dynamic face localizer task and repetition suppression parameter estimates extracted from the FFA. ***A***, The spatial extent of the key ROIs of interest, the FFA, and the OFA, presented as degree of spatial overlap between participants in normalized space. We generated these ROIs with the group-constrained subject-specific (GcSS) approach (Julian et al., 2012) for the contrast of faces > other categories at the level of each individual participant. For a table listing the full ROI list, see Extended Data Figure 3-6. ***B***, Repetition suppression parameter estimates in the FFA for different-face and repeated-face pairs for Black and White male faces. Upper panel: to present repetition suppression effects, we subtracted neural activity in response to single faces (baseline) from the neural response to the different conditions (differ, repeat), separately for each race (Black, White). Negative values indicate neural suppression compared with baseline. Across all figures, individual dots represent neural suppression for unique participants. Each figure also visualizes the mean of each condition (as a red dot), the median (solid horizontal line), and first and third quartiles (boxplot). Lower panel: mean effect size (the difference in suppression effect) and the bootstrapped 95% confidence intervals for the comparison between Black and White targets in each condition. The results demonstrate more release from suppression (i.e., less negative values) for different White targets compared with different Black targets, but no difference in suppression for repeating Black and White targets. This pattern suggests that the FFA forms unique representations for different individuals, but only for members of one’s own racial group. We report the results of the parallel analysis in the OFA and visual cortex in Figure 4. Extended Data Figure Figures 3-1, 3-2, 3-3, 3-4 demonstrate the robustness of the effect to different analytic strategies. In Extended Data Figure 3-5, we report the results of the main analysis with female faces.

Participants in experiment 1 demonstrated a behavioral outgroup homogeneity effect in the repetition paradigm. To examine neural representation differences between the groups, experiment 2 included 29 White participants who performed the task while undergoing fMRI scanning. We used a separate face localizer task (Pitcher et al., 2011) to identify a priori ROIs in right fusiform gyrus (FFA) and right occipital cortex (OFA; Fig. 3*A*). We did not identify a robust cluster in a third hypothesized ROI, the anterior temporal lobe (for details of localization procedure, see Materials and Methods; Julian et al., 2012).

**Figure 4.**
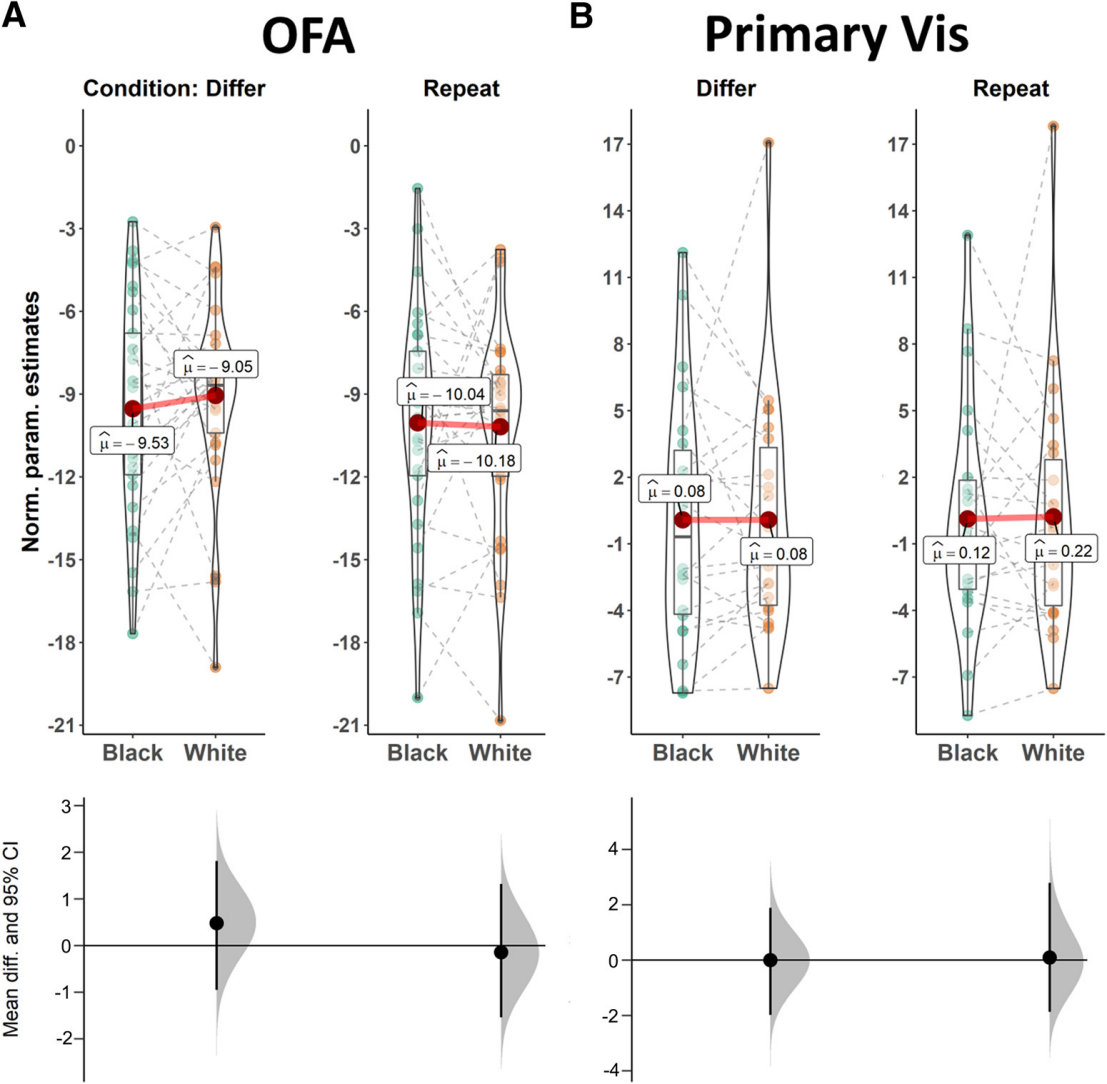
Repetition suppression results for repeated and different Black and White targets for the OFA and primary visual cortex (for additional details on analysis approach, see Materials and Methods). Similar to Figure 3*B*, negative values indicate neural suppression compared with baseline. The lower panel depicts effect size estimate (the difference in suppression effect) and the bootstrapped 95% confidence intervals for the comparison between Black and White targets in each condition. ***A***, The OFA demonstrated robust repetition suppression for repeated and different faces (*F*_(1,28)_ = 245.88, MSE = 12.06, *p *<* *0.001, η^2^_p_ = 0.90 and *F*_(1,28)_ = 247.03, MSE = 10.13, *p *<* *0.001, η^2^_p_ = 0.90 for repeated and different targets, respectively). The OFA showed some release from suppression for different faces (*F*_(1,28)_ = 9.93, MSE = 1.98, *p *=* *0.004, η^2^_p_ = 0.26), but we found no indication for an effect of race on suppression for repeated or different targets (*F*_(1,28)_ = 0.04, MSE = 3.88, *p *=* *0.85, η^2^_p_ = 0.001 and *F*_(1,28)_ = 0.46, MSE = 3.62, *p *=* *0.50, η^2^_p_ = 0.02, respectively). Thus, the OFA was not sensitive to the group features of the different faces. ***B***, Primary visual cortex (including V1) served as a control condition. No effects were observed in this region (all *F*s < 1.4, all *p*s* *>* *0.2).

10.1523/ENEURO.0431-19.2020.f3-1 Extended Data Figure 3-1 Repetition suppression parameter estimates for the FFA, OFA, and primary visual cortex for analyses including participants we excluded in the main text (*N* = 32). Upper panel, Repetition effects for all individuals. Lower panel, Effect size estimate and the bootstrapped 95% confidence intervals for the comparison between Black and White targets in each condition. Overall, results replicate with this sample (compare to Figs. 3*B*, 4). ***A***, FFA results. Activation in FFA was suppressed to nearly an equivalent degree for both Black and White repeated targets [repetition effect: *F*_(1,31)_ = 167.86, MSE = 5.32, *p *<* *0.001, η^2^_p_ = 0.84; interaction effect: *F*_(1,31_ = 1.50, MSE = 0.98, *p *=* *0.23, η^2^_p_ = 0.05; equivalence test for the two repetition effects (mean difference: 0.43): *p* = 0.056, equivalence interval (–1.02, 0.16)]. FFA also showed more release from suppression for White targets than for Black targets (*F*_(1,31)_ = 11.96, MSE = 0.56, *p *=* *0.002, η^2^_p_ = 0.31; interaction between repeated and different faces: *F*_(1,31)_ = 3.35, MSE = 0.57, p = 0.08, η^2^_p_ = 0.10). And again, similar to the main findings, we observed statistically equivalent levels of repetition suppression for different and repeated Black targets [equivalence test (mean difference: 0.46): *p = *0.005, (–0.80, –0.12)]. ***B***, The OFA demonstrated robust repetition suppression for repeated and different faces (*F*_(1,31)_ = 273.17, MSE* *=* *11.83, *p *<* *0.001, η^2^_p_ = 0.90 and *F*_(1,31)_ = 261.09, MSE* *=* *10.37, *p *<* *0.001, η^2^_p_ = 0.89 for repeated and different trials, respectively). The OFA showed some release from suppression for different faces (*F*_(1,31)_ = 12.43, MSE* *=* *1.87, *p *=* *0.001, η^2^_p_ = 0.29), but no indication for an effect of race on any of these results (*F*_(1,31)_ = 0.05, MSE = 3.59, *p *=* *0.83, η^2^_p_ = 0.002 and *F*_(1,31)_ = 0.28, MSE =* *3.39, *p *=* *0.50, η^2^_p_ = 0.009 for repeated and different targets, respectively). Thus, the OFA was not sensitive to the group features of the different faces. ***C***, We did not observe any effects in the primary visual cortex, again replicating the principal analysis (all *F*s < 2.02, all *p*s > 0.17). Download Figure 3-1, TIF file.

10.1523/ENEURO.0431-19.2020.f3-2 Extended Data Figure 3-2 Repetition suppression parameter estimates for the FFA, OFA, and primary visual cortex for analyses excluding targets that participants incorrectly classified (preregistered complementary analysis; *N* = 29). Upper panel, Repetition effects for all individuals. Lower panel, Effect size estimate and the bootstrapped 95% confidence intervals for the comparison between Black and White targets in each condition. As with the previous analysis, results replicate the main findings. ***A***, FFA results. Activation in FFA was suppressed to nearly an equivalent degree for both Black and White repeated faces [repetition effect: *F*_(1,28)_ = 180.11, MSE* *=* *4.56, *p *<* *0.001, η^2^_p_ = 0.87; interaction effect: *F*_(1,28)_ = 0.28, MSE* *=* *1.60, *p *=* *0.60, η^2^_p_ = 0.01; equivalence test for the two repetition effects (mean difference: 0.25): *p* = 0.0598, equivalence interval (–1.05, 0.55)]. FFA also showed more release from suppression for White targets than for Black targets (*F*_(1,28)_ = 6.94, MSE* *=* *0.60, *p *=* *0.01, η^2^_p_ = 0.20; interaction between repeated and different faces: *F*_(1,28)_ = 2.01, MSE* *=* *0.94, *p *=* *0.17, η^2^_p_ = 0.07]. And again, similar to the main findings, we observed statistically equivalent levels of repetition suppression for different and repeated Black targets [equivalence test (mean difference: 0.47): *p = *0.036, (–0.95, 0.02)]. ***B***, The OFA demonstrated robust repetition suppression for repeated and different faces (*F*_(1,28)_ = 233.52, MSE* *=* *12.73, *p *<* *0.001, η^2^_p_ = 0.89 and *F*_(1,28)_ = 267.83, MSE* *=* *9.52, *p *<* *0.001, η^2^_p_ = 0.91 for repeated and different trials, respectively). The OFA showed some release from suppression for different faces (*F*_(1,28)_ = 4.70, MSE = 3.46, *p *=* *0.04, η^2^_p_ = 0.14). As before, race did not interact with suppression for repeated or different faces (*F*_(1,28)_ = 0.99, MSE = 7.52, *p *=* *0.33, η^2^_p_ = 0.03 and *F*_(1,28)_ = 0.08, MSE = 4.51, *p *=* *0.78, η^2^_p_ = 0.003 for repeated and different targets, respectively). Thus, once again, the OFA was not sensitive to the group features of the different faces. ***C***, We did not observe any effects in the primary visual cortex, again replicating the principal analysis (all *F*s < 1.13, all *p*s* *>* *0.30). Download Figure 3-2, TIF file.

10.1523/ENEURO.0431-19.2020.f3-3 Extended Data Figure 3-3 Parameter estimates for the FFA, OFA, and primary visual cortex for the original preregistered analysis, in which we erroneously modelled single target trials with variable durations based on participants’ reaction time to the scrambled image that followed the single face (*N* = 29). Upper panel, Repetition effects for all individuals. Lower panel, Effect size estimate and the bootstrapped 95% confidence intervals for the comparison between Black and White targets in each condition. As in all previous analyses, we plot neural activity by subtracting the response to single faces (baseline) from the neural response to the different conditions (different, repeated), separately for each race (Black, White). Parameter estimates in this analysis are positive and no longer reflect suppression, as the baseline in this analysis reflects the neural activity in response to the scrambled image, rather than an actual face. This response was naturally weaker in face-sensitive regions. Nonetheless, overall pattern of results replicated the principal analysis. ***A***, FFA results. Activation was different from baseline to an equivalent degree for Black and White repeated faces [repetition effect: *F*_(1,28)_ = 51.79, MSE* *=* *0.35, *p *<* *0.001, η^2^_p_ = 0.65; interaction effect: *F*_(1,28)_ = 0.15, MSE* *=* *0.50, *p *=* *0.70, η^2^_p_ = 0.005; equivalence test for the two repetition effects (mean difference: 0.10): *p* = 0.001, equivalence interval (–0.55, 0.34)]. FFA also showed more difference from baseline for White targets than for Black targets (*F*_(1,28)_ = 6.74, MSE* *=* *0.31, *p *=* *0.01, η^2^_p_ = 0.19; interaction between repeated and different faces: *F*_(1,28)_ = 3.65, MSE* *=* *0.38, *p *=* *0.07, η^2^_p_ = 0.12). And again, similar to the main findings, we observed statistically equivalent levels of difference from baseline for different and repeated Black targets [equivalence test (mean difference: 0.28): *p < *0.001, (–0.55, –0.003)]. ***B***, The OFA demonstrated difference from baseline only for different and not for repeated faces (*F*_(1,28)_ = 2.48, MSE* *=* *1.48, *p *=* *0.13, η^2^_p_ = 0.08 and *F*_(1,28)_ = 28.66, MSE* *=* *1.26, *p *<* *0.001, η^2^_p_ = 0.51 for repeated and different trials, respectively). The difference between OFA response to different and repeated faces was significant (*F*_(1,28)_ = 8.67, MSE* *=* *1.94, *p *=* *0.006, η^2^_p_ = 0.24). As before, race did not interact with difference from baseline for repeated or different faces (*F*_(1,28)_ = 0.55, MSE* *=* *1.42, *p *=* *0.47, η^2^_p_ = 0.02 and *F*_(1,28)_ = 0.99, MSE* *=* *1.26, *p *=* *0.33, η^2^_p_ = 0.03, respectively). Thus, the OFA was not sensitive to the group features of the different faces. ***C***, We did not observe any effects of race or interaction with race in the primary visual cortex, again replicating the principal analysis (all *F*s < 2.13, all *p*s > 0.16). Download Figure 3-3, TIF file.

10.1523/ENEURO.0431-19.2020.f3-4 Extended Data Figure 3-4 Parameter estimates in the (***A***) FFA and (***B***) OFA for the three experimental conditions for Black and White male faces (without correcting for differences in baseline activity). Upper panels, Neural activity in response to the different conditions (single, different, repeated), separately for each race (Black, White). Individual dots represent neural activity for unique participants. Vertical lines (presented in parallel to the scatter plot) depict the mean and SD for each condition. Lower panel, Mean effect size (the repetition suppression effect) and the bootstrapped 95% confidence intervals for the comparison between type of repetition and single-face targets for each race. For statistical analyses, see Figure 3 and main text. Download Figure 3-4, TIF file.

10.1523/ENEURO.0431-19.2020.f3-5 Extended Data Figure 3-5 Repetition suppression results for female targets for the FFA, OFA, and primary visual cortex using the analysis reported in the main manuscript (compare to results for male targets in Figs. 3*B*, 4). We subtracted neural activity in response to single faces (baseline) from the neural response to the different conditions (different, repeated), separately for each race (Black, White; upper panel). The lower panel depicts effect size estimate (the difference in suppression effect) and the bootstrapped 95% confidence intervals for the comparison between Black and White targets in each condition. ***A***, The FFA demonstrated robust repetition suppression for repeated and different faces (*F*_(1,28)_ = 392.66, MSE = 2.48, *p *<* *0.001, η^2^_p_ = 0.93 and *F*_(1,28)_ = 290.91, MSE* *=* *2.66, *p *<* *0.001, η^2^_p_ = 0.91 for repeated and different trials, respectively). The FFA also demonstrated release from suppression for different faces (*F*_(1,28)_ = 14.66, MSE* *=* *0.78, *p *<* *0.001, η^2^_p_ = 0.34), an effect that was qualified by an interaction with race (*F*_(1,28)_ = 6.76, MSE* *=* *0.38, *p *=* *0.01, η^2^_p_ = 0.19). However, the FFA did not show significant simple effects (i.e., differences in suppression) between Black and White female targets (*F*_(1,28)_ = 2.41, MSE* *=* *1.58, *p *=* *0.13, η^2^_p_ = 0.08 and *F*_(1,28)_ = 0.08, MSE* *=* *1.51, *p *=* *0.77, η^2^_p_ = 0.003 for repeated and different faces, respectively). As we did not have an a priori hypothesis for female targets, we cannot offer a reliable interpretation of these results. ***B***, Much like for male targets, the OFA demonstrated robust repetition suppression for repeated and different faces (*F*_(1,28)_ = 517.23, MSE* *=* *6.92, *p *<* *0.001, η^2^_p_ = 0.95 and *F*_(1,28)_ = 371.93, MSE* *=* *8.28, *p *<* *0.001, η^2^_p_ = 0.93 for repeated and different trials, respectively). The OFA showed some release from suppression for different faces (*F*_(1,28)_ = 6.70, MSE* *=* *2.79, *p *=* *0.02, η^2^_p_ = 0.19), but no indication of an effect of race on any of these results (*F*_(1,28)_ = 3.25, MSE* *=* *4.84, *p *=* *0.08, η^2^_p_ = 0.10 and *F*_(1,28)_ = 2.08, MSE* *=* *4.71, *p *=* *0.16, η^2^_p_ = 0.07 for repeated and different targets). Thus, as for male targets, the OFA was not sensitive to the group features of the different female faces. ***C***, Primary visual cortex (including V1) results. No effects were observed in this region (all *F*s < 1.2, all *p*s* *>* *0.2). Overall, we did not observe any neural evidence for the outgroup homogeneity effect for female targets, paralleling the behavioral results. Download Figure 3-5, TIF file.

10.1523/ENEURO.0431-19.2020.f3-6 Extended Data Figure 3-6 Results from the main functional ROI analysis [group-constrained subject-specific (GcSS); see Materials and Methods]. For each ROI, the GcSS algorithm defines a parcel to intersect with activation for each individual participant, thus reliably identifying the same functional ROIs in all participants. We conducted the GcSS analyses with the contrast of faces > other categories in independent localizer scans. A priori ROIs are presented in italics. Download Figure 3-6, DOCX file.

We then examined patterns of repetition suppression for repeated and different faces. For each condition of interest (repeated and different faces), we computed the differences from baseline (single face) for Black and White targets. In line with our preregistration and experiment 1, we report the results for male targets only (for results for female targets, see Extended Data Fig. 3-5). When a second face was identical to the first, activation in FFA was suppressed to an equivalent degree for both Black and White faces [repetition effect: *F*_(1,28)_ = 160.20, mean squared error (MSE)* *=* *4.83, *p *<* *0.001, η^2^_p_ = 0.85; interaction effect: *F*_(1,28)_ = 1.34, MSE* *=* *0.82, *p *=* *0.26, η^2^_p_ = 0.05; equivalence test for the two repetition effects (mean difference: 0.39): *p = *0.04, equivalence interval (−0.96, 0.18)]. In other words, perceivers showed similar levels of suppression in FFA when faces were repeated, regardless of race. By contrast, FFA was released from suppression when the second face differed from the first, but only for White faces. We observed more release from suppression for White faces than for Black faces, suggesting that participants perceived White faces, but not Black faces, as representing different individuals (repetition effect: *F*_(1,28)_ = 13.16, MSE* *=* *0.57, *p *=* *0.001, η^2^_p_ = 0.32; interaction between repeating and different faces: *F*_(1,28)_ = 7.64, MSE* *=* *0.37, *p *=* *0.01, η^2^_p_ = 0.21; Fig. 3*B*). Remarkably, we observed similar levels of repetition suppression for two different Black individuals as for two identical faces [equivalence test for suppression of different and repeated Black faces (mean difference: 0.24): *p < *0.001, (−0.52, 0.04)], which suggests that participants did not consistently perceive the two photographs to represent distinct individuals.

This pattern of results was selective for the FFA, which is thought to be the earliest visual area that encodes the unique identity of faces, rather than just their distinct perceptual features (Duchaine and Yovel, 2015). Patterns of repetition suppression did not vary for different Black and White faces in either the OFA (*F*_(1,28)_ = 0.46, MSE* *=* *3.62, *p *=* *0.50, η^2^_p_ = 0.02) or primary visual cortex (*F*_(1,21)_ < 0.01, MSE* *=* *5.28, *p *>* *0.99, η^2^_p_ < 0.001; Fig. 4). Finally, results were robust across several preregistered analytic variations, including analyses with excluded participants as well as when excluding trials that were answered incorrectly (see Materials and Methods; Extended Data Figs. 3-1, 3-2, 3-3). Together, these findings suggest that the FFA (but not lower-level visual regions) processes racial outgroup individuals as more similar to each other relative to ingroup individuals.

The present results expand on previous investigations in several important ways. First, most studies of the other-race or the other-group effect have documented increased univariate activity in the FFA in response to own-group faces compared with faces from a different social group (Golby et al., 2001; Kim et al., 2006; Van Bavel et al., 2008, 2011; Feng et al., 2011; for review, see Molenberghs and Louis, 2018). However, mean activity level can be susceptible to multiple moderators, including attention and motivation. Indeed, a recent study demonstrated that participants who experience resource scarcity demonstrate reduced FFA activity for Black faces and increased FFA activity for White faces (Krosch and Amodio, 2019). Thus, mean FFA activity can reflect the influence of contextual factors on face perception, rather than measure the representations underlying the perceived face. Here, instead of analyzing mean activity, we use the robust phenomenon of repetition suppression to demonstrate that the FFA utilizes a similar representation for different outgroup, but not ingroup, faces.

Our approach also complements a recent demonstration of differential release from suppression for Black and White targets (Hughes et al., 2019). One outstanding issue concerns the demonstration of the repetition effect. In their study, Hughes and colleagues compared blocks of repeated faces to blocks of faces morphed to different degrees, which assumes equivalent magnitude of neural activity for repeated White and Black faces without explicitly accounting for it. By contrast, our paradigm allowed us to quantify neural activity for baseline, repeated, and different face trials for Black and White faces separately, providing a straightforward index of repetition suppression and release from suppression. Second, we observed behavioral and neural outgroup homogeneity effects within the same paradigm, whereas Hughes and colleagues demonstrated neural and behavioral effects in disparate paradigms. Third, we adopted a subject-specific ROI selection approach and included exploratory analyses of female faces. Thus, our preregistered study provides a substantial extension of the existing literature by (1) conceptually replicating prior work (Hughes et al., 2019), thereby bolstering our confidence in the reliability of this effect; and (2) providing further evidence for representation-based accounts of the other-race homogeneity effect (see also Yaros et al., 2019). Notably, neither of these findings speak to the developmental origins of the effect. Specifically, individuals continuously absorb information from their environment, and their social behaviors and representations undoubtedly update as a function of this input (Rule et al., 2013). Thus, our findings cannot indicate whether the differences we observed stem from innate processes or, alternatively, were acquired throughout participants’ lifetimes.

Our findings have two potential limitations. First, we observed the effects only for male and not for female targets (Fig. 3; Extended Data Fig. 3-5). One possibility is that women are less likely to be targets of the outgroup homogeneity effect. These findings accord with a broader literature documenting that outgroup men are more likely targets of intergroup discrimination and harm than are women [e.g., social dominance theory (Sidanius and Pratto, 2001) and the theory of gendered prejudice (Sidanius et al., 2019), both highlighting the importance of gender as a moderator in intergroup relations]. These findings could also indicate that representations in the FFA reflect multiple categorical geometries, including race, gender, and their interaction (Freeman et al., 2018). That said, we found that images of Black female targets in our task were more different from each other (with regard to image properties) than the other conditions. Thus, a third possibility is that increased variability made different Black female faces more distinguishable. Rather than omit female faces from the paradigm like so many other studies of this phenomenon, the current investigation included female targets to help start building a knowledge base to adjudicate among these competing explanations. A second limitation of our study is that it was constrained to White perceivers; nevertheless, given the robustness of the outgroup homogeneity effect across groups and cultures (Wan et al., 2015; Kokje et al., 2018), these results are likely to generalize to additional groups and targets (e.g., Asian perceivers viewing White faces). Testing this hypothesis remains a goal for future studies.

Many theories of human sociality begin with the assumption that perceivers can keep track of others’ reputation by correctly identifying and later remembering what they did, and to whom they did it. Nevertheless, humans routinely fail to engage in such basic social cognition for outgroup members, in large part because perceivers do not consistently distinguish among individual members of such outgroups (especially those delineated by race and ethnicity). This outgroup homogeneity effect undermines one of the basic starting conditions of human (pro)sociality, in that it forestalls the ability to identify individuals with a unique set of past actions and behavioral tendencies. Here, we suggest that the potential origins of the outgroup homogeneity effect lie in failures of visual processing to form distinct representations of individual members of outgroups, something that it nevertheless accomplishes exquisitely for members of one’s own social groups.

## Synthesis

Reviewing Editor: Daniela Schiller, Mount Sinai School of Medicine Decisions are customarily a result of the Reviewing Editor and the peer reviewers coming together and discussing their recommendations until a consensus is reached. When revisions are invited, a fact-based synthesis statement explaining their decision and outlining what is needed to prepare a revision will be listed below.

Advances to the field:

Provides novel contributions to a relatively sparse literature.

While the results presented largely replicate prior work, this still represents an incremental advance for the field, as this consistency allows for added confidence in the results observed across investigations of this phenomenon.

Statistics:

Some of the statistical approaches appear to be inconsistent across experiments. It is also unclear to me that the use of the baseline condition in the fMRI experiment is optimal. That being said, it is possible that further justifications for these choices are in the pre-registration document.

The figures should indicate what the error bard indicate.

For all figures - it is recommended to use estimation statistics (including individual observed values, confidence intervals, effect size, precision of effect size, confidence and likelihood of effect size intervals).

Extended data:

These extended data present additional pre-registered analysis approaches and serve to rule out alternative explanations concerning the main results.

Software comments:

SPM12 and R were mentioned, but it’s not clear to me what it would mean to test and validate these software packages (e.g., they are widely used for the purposes described in this manuscript). All external packages and approaches seem to be appropriately cited.

Overall comments:

Review #1

The authors present the results of two experiments - one behavioral and one neuroimaging investigation - concerning the outgroup homogeneity effect. They convincingly demonstrate (via a repetition suppression paradigm) that face-selective areas in inferotemporal cortex associated with processing identity show greater adaptation to consecutive images of the different black targets, versus consecutive images of different white targets. This is interpreted as failure to individuate black (vs. white) targets in the context of this task. These data are supported by behavioral findings showing reductions in other-race face memory in the same paradigm.

In evaluating this manuscript, I tried to take into account its novelty, its quality, and the value of replication as separate factors. As the authors note, there has been previous work published along these same lines already: “...the FFA should show more suppression when a Black face is followed by a new, different Black face despite, in fact, the second face being different from the first (for related approaches, see Hughes et al., 2019; Vizioli, Rousselet, & Caldara, 2010).” The work from Hughes et al. (2019) is especially relevant as compares repetition suppression between white and black targets.

In my view, there is equal value in replication and novelty. The effects observed in the present manuscript fall in line remarkably with those presented by Hughes et al. (2019). Given concerns regarding the replicability of results in human neuroimaging work (and social neuroscience in particular), a replication this direct and clean is impressive and important-especially given a research topic of such social consequence. (I recognize that this data was likely collected contemporaneous to the Hughes et al. work, so I use “replication” to refer broadly to the consistency of results across the two studies, not a concerted effort to replicate the earlier work.)

That said, it would be extremely helpful if the authors could provide *some* commentary on how the present data expand on these previous investigations, or whether they address any gaps or flaws in that work - if at all possible. Moreover, I think the manuscript would benefit from highlighting the consistency of these results more directly.

I have a few follow-up suggestions and questions:

The authors suggest that due to the FFA’s involvement in processing identity (e.g., Duchaine & Yovel, 2015), these results suggest that “the outgroup homogeneity effect is linked to failures of early visual processing to form distinct representations of individual members of outgroups.” The word “early” is used throughout the text to describe different aspects of a processing stream for faces (reflected in the revised model depicted in the Duchaine & Yovel review; e.g., Fig. 7), but at different times is applied to brain regions generally involved in visual perception (e.g., “early visual cortex”) and at other times is applied to brain regions involved specific to face perception (e.g., in the final line of the paper). (Another mention in the abstract describes “early-to-mid-level visual processing.”) Could this be more consistent throughout the text

What was the race of the faces used in the dynamic localizer task? If these faces are racially homogenous (particularly if they’re all white), does this pose concerns regarding drawing inferences from the fusiform ROI localized via this task?

Was hair cropped out of the face stimuli? (Presumably this may influence the gender X race interaction in terms of image similarity on pg. 7.)

The authors describe an extensive procedure used to select faces that were matched in terms of their racial prototypicality. That said, the CFD offers a host of norming data regarding other attributes of these faces (e.g., attractiveness, dominance, trustworthiness, latent emotional content). Were the subsets selected balanced on these dimensions? If not, does it constrain the inferences that can be made from the results, if, say, the white faces are rated as looking less dominant, or there’s a smaller range for trustworthiness ratings of black faces?

Regarding power analysis, the authors state, “We identified this sample size to be sufficient to achieve a power 89 of 0.8 to detect a hypothesized meaningful effect size estimate of 0.3 for the interaction contrast of race (Black/White) by condition (repeated/different versus single).” What metric of effect size are the authors using here? (e.g., Cohen’s d? r? something else?)

Power for investigating brain-to-behavior correlations is low, but did the authors observe a relationship between individual differences in the black-white repetition suppression difference score and task performance in Exp. 2?

Review #2

The authors reported two experiments demonstrating behavioral outgroup homogeneity effect and, using fMRI, greater repetition suppression effects in FFA for Black Male faces even when in conditions where the faces were not repeated. In Experiment 1, behavioral results showed White perceivers were slower to identify repeated faces and subsequently less accurate at recognizing Black males relative to White males. In Experiment 2, fMRI results showed that the face-sensitive FFA was released from suppression more for White male targets than for Black male targets. Interestingly, this pattern of result was not obtained in OFA or early visual cortex. In both Experiments 1 and 2 these effects were found among male targets but not female targets.

I believe this is a well-designed study that will contribute to the literature on race perception and may highlight some mechanisms underlying the outgroup homogeneity effect. However, this contribution may be limited by the sparse review of the relevant literatures (both in terms of neuroscience of race perception and social cognitive investigations of outgroup homogeneity) and the very concise discussion. For example, some of the novelty of these findings comes from the different results for male and female targets. I can’t access the preregistration but if the gender differences were predicted, this prediction should probably be explained in the introduction. If the gender differences were not predicted, I still think at least some explanation of why target gender was included as an exploratory factor would be useful. Furthermore, the current discussion of these findings (that they are consistent with social dominance theory (Sidanius & Pratto, 2001) and the theory of gendered prejudice (Sidanius, Hudson, Davis, & Bergh, 2019)) seem insufficient and somewhat contradictory to the premise of the article (that the outgroup homogeneity effects are based on differences in perceptual representations of racial outgroup members which interferes with individuation processes.

Some of the stated contributions of the findings seem to be somewhat disconnected from the results. When the researcher claim the following:”Researchers have repeatedly demonstrated that perceivers struggle to distinguish between different members of a racial outgroup. Here, we show in a pre-registered study that this failure arises when areas of the human brain that specifically process facial identity-most notably, the so-called “fusiform face area"-fail to detect differences between identities of members of a racial outgroup.” We would expect that that the observed neural repetition suppression effects were tied to the be behavioral outgroup homogeneity effects but the authors do not present data suggesting this is the case and furthermore provide analyses suggesting that it may not be the case. While it may indeed be possible to identify such relationship in a study with many more trials, I think it’s important not to suggest this is the case here. Accordingly, I believe the manuscript would benefit from some restructuring.

Additional points:

I’m confused about the stimulus counts on lines 119-121. Why were there 76 acceptable stimuli per condition from the CFD in the fMRI study (Experiment 2) but only 72 in the behavioral study (Experiment 1)?

I would like to see some interpretation of the SSIM analysis results (lines 126-133). The SSIM analysis was presented to rule out image similarity as a confound, but the results differed by gender: Black men were as structurally similar as White men, but Black women were more structurally dissimilar than White women. It is not immediately obvious to me how to interpret this in the context of the current findings.

I am not sure why the statistical analyses for Experiment 1 included target gender as a factor, but the statistical analyses for Experiment 2 seems to have split the data by target gender (lines 261-262, “The model included six conditions per gender (2 races by 3 conditions: repeated faces, different faces, single face).”) Can the authors explain this change in the statistical approach?

It is not clear until the paragraph on limitations that the effects for Experiment 2 are also only for the male targets. I would prefer seeing that highlighted more clearly at the beginning of the description of the results for Experiment 2.

In Experiment 2, it would be helpful to explain the baseline in the main text of the results section instead of only in the figure captions.

I also think it could be valuable to add in supplements ROI analyses that do not include subtraction of the “baseline trials”. For instance, I wonder if the relative perceptual salience of the Black and White faces may in part explain the differences in result between the FFA and OFA.

I imagine there isn’t sufficient power to look at perceiver gender as a factor in these Experiments, but I wonder if that might be a useful future direction to mention?
