# Supplementary figures and images for: Human Face-Selective Cortex Does Not Distinguish between Members of a Racial Outgroup
Source: eNeuro. 2020 May 29;7(3):ENEURO.0431-19.2020. doi: 10.1523/ENEURO.0431-19.2020 (PMC7266143; doi:10.1523/ENEURO.0431-19.2020)

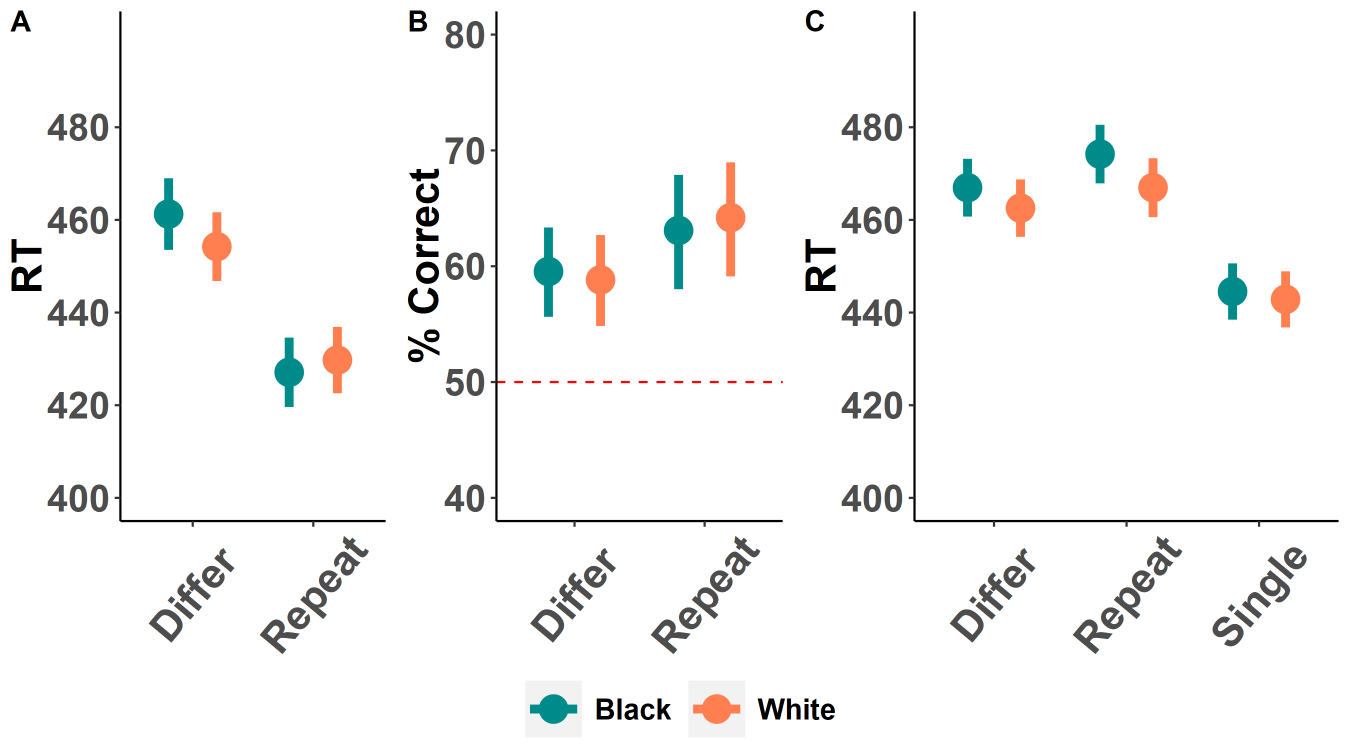

Supplement: Extended Data Figure 1-1 — Reaction time and memory performance for female faces in experiments 1 and 2. All panels depict estimates computed by gLMMs, as detailed in Materials and Methods. A, Participants in experiment 1 were faster to respond to repeated faces than to different faces (t = 8.43, p < 0.001). However, unlike responses for male faces, there was no evidence for an effect of race (t = 0.34, p = 0.74) nor for an interaction of race with type of repetition (interaction model comparison: χ(1) = 1.93, p = 0.16, suggesting that a model with no interaction term provides a better fit to the data). This pattern hints that Black female faces were individuated to the same extent as White female faces. When we included all trials to test a full model of the data, with the fixed effects of race, repetition, and gender, we observed a significant three-way interaction (model comparison: χ(4) = 10.67, p = 0.031), confirming the difference in individuation between male and female faces. B, Unlike male faces, participants were not more likely to remember White female faces compared to Black female faces (OR = 1.004, Wald’s z = 0.09, p = 0.93). Participants were more likely to remember repeated faces compared to different faces (OR = 1.10, Wald’s z = 2.20, p = 0.028) with no interaction with race (OR = 1.02, Wald’s z = 0.456, p = 0.65). However, we did not observe a statistically significant three-way interaction effect for gender when it was inserted into the model (χ(4) = 6.88, p = 0.14). C, Similar to responses to male faces, participants in experiment 2 responded faster to single face trials (model comparison: χ(2) = 111.42, p < 0.001). However, unlike response times for male faces, we did not observe a robust effect of race (t = 1.97, p = 0.049) nor an interaction of race and repetition (model comparison: χ(2) = 1.03, p = 0.597; we obtained similar results when omitting the single condition from the analysis). As in experiment 1, when we included the fixed effects of race, repetition, and gen [file enu-eN-CFN-0431-19-s01.tif]

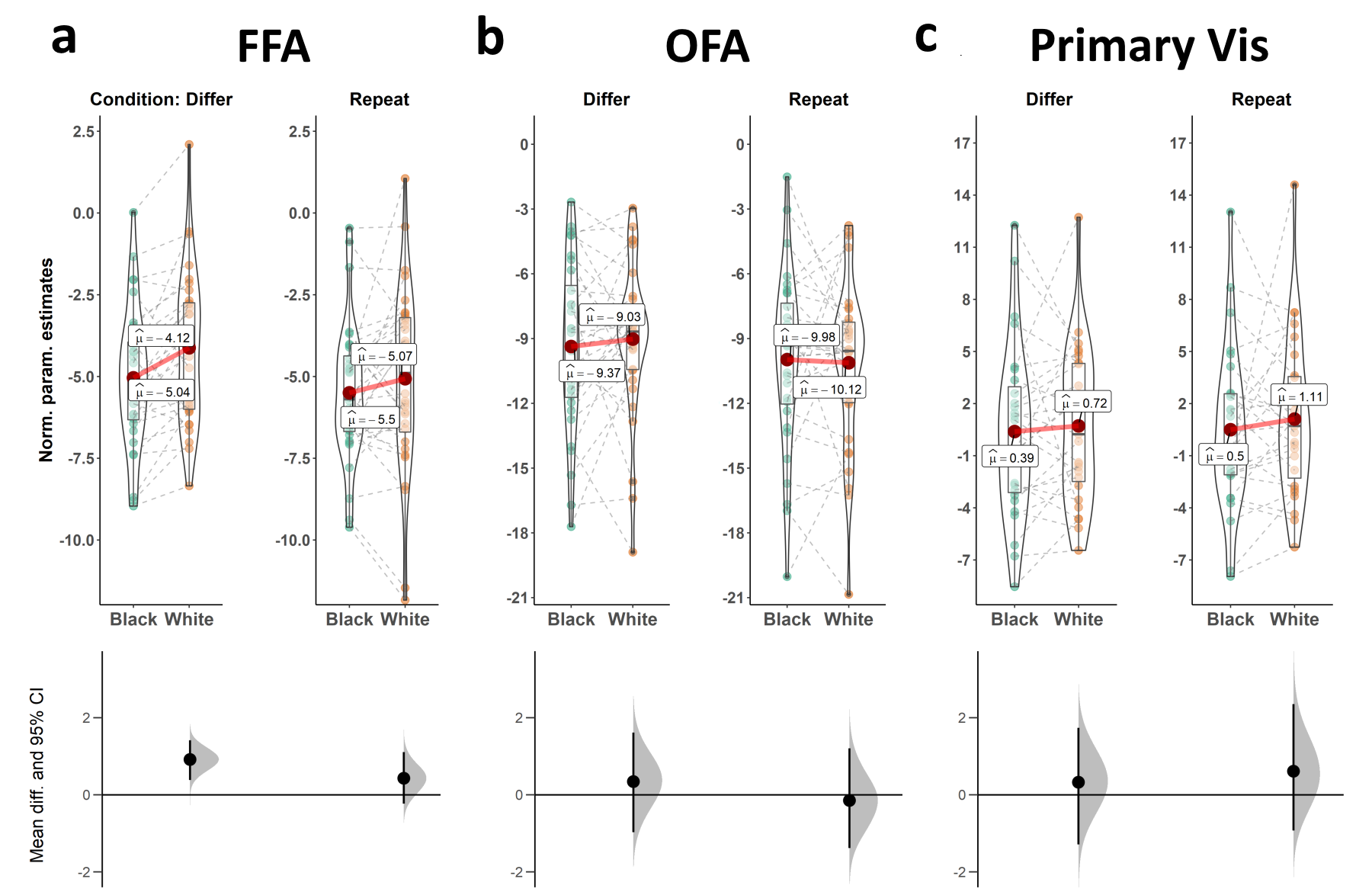

Supplement: Extended Data Figure 3-1 — Repetition suppression parameter estimates for the FFA, OFA, and primary visual cortex for analyses including participants we excluded in the main text (N = 32). Upper panel, Repetition effects for all individuals. Lower panel, Effect size estimate and the bootstrapped 95% confidence intervals for the comparison between Black and White targets in each condition. Overall, results replicate with this sample (compare to Figs. 3B, 4). A, FFA results. Activation in FFA was suppressed to nearly an equivalent degree for both Black and White repeated targets [repetition effect: F(1,31) = 167.86, MSE = 5.32, p < 0.001, η2p = 0.84; interaction effect: F(1,31 = 1.50, MSE = 0.98, p = 0.23, η2p = 0.05; equivalence test for the two repetition effects (mean difference: 0.43): p = 0.056, equivalence interval (–1.02, 0.16)]. FFA also showed more release from suppression for White targets than for Black targets (F(1,31) = 11.96, MSE = 0.56, p = 0.002, η2p = 0.31; interaction between repeated and different faces: F(1,31) = 3.35, MSE = 0.57, p = 0.08, η2p = 0.10). And again, similar to the main findings, we observed statistically equivalent levels of repetition suppression for different and repeated Black targets [equivalence test (mean difference: 0.46): p = 0.005, (–0.80, –0.12)]. B, The OFA demonstrated robust repetition suppression for repeated and different faces (F(1,31) = 273.17, MSE = 11.83, p < 0.001, η2p = 0.90 and F(1,31) = 261.09, MSE = 10.37, p < 0.001, η2p = 0.89 for repeated and different trials, respectively). The OFA showed some release from suppression for different faces (F(1,31) = 12.43, MSE = 1.87, p = 0.001, η2p = 0.29), but no indication for an effect of race on any of these results (F(1,31) = 0.05, MSE = 3.59, p = 0.83, η2p = 0.002 and F(1,31) = 0.28, MSE = 3.39, p = 0.50, η2p = 0.009 for repeated and different targets, respectively). Thus, the OFA was not sensitive to the group features of the different faces. C, We did not observe any effects in the primary vi [file enu-eN-CFN-0431-19-s02.tif]

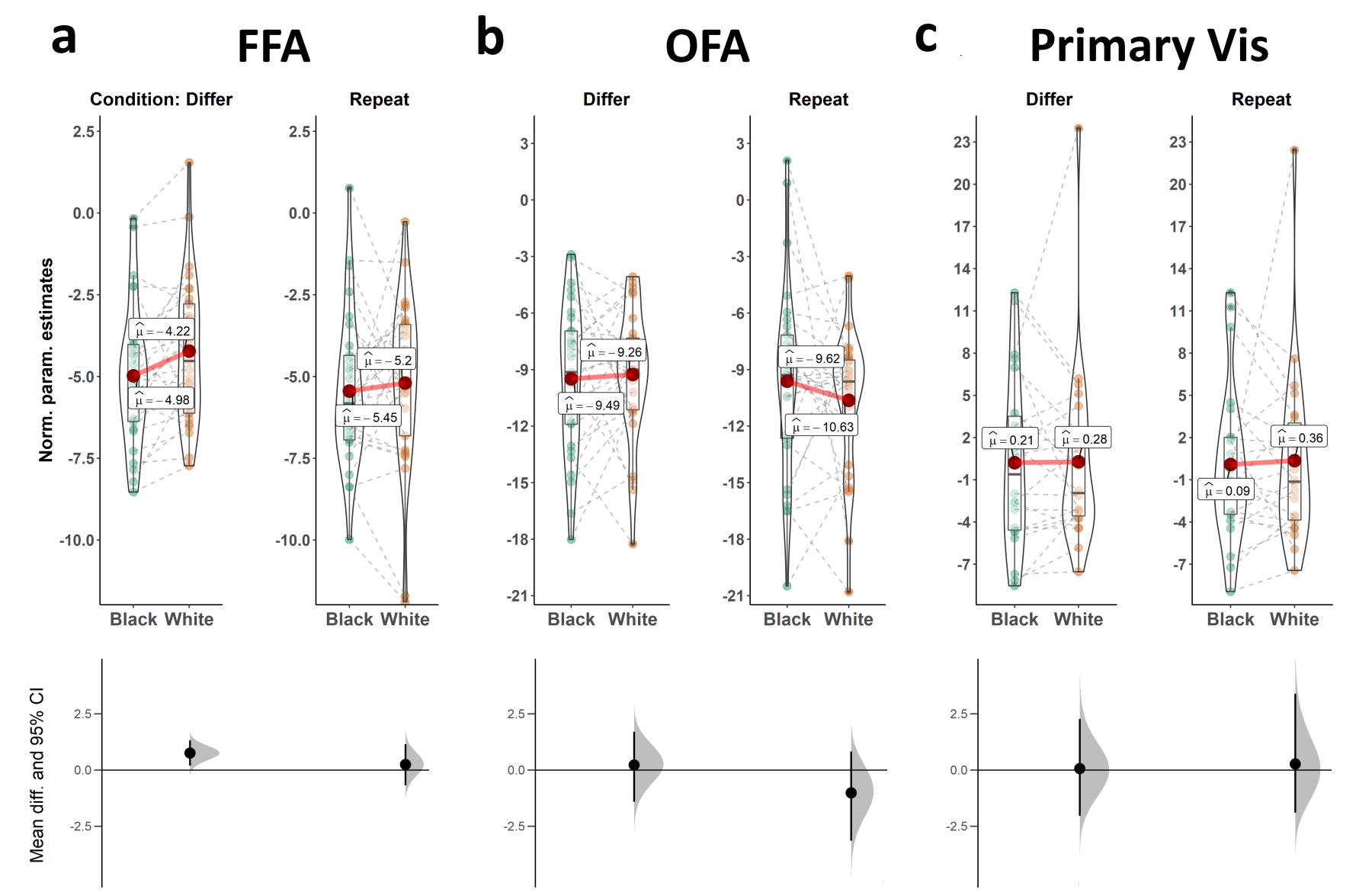

Supplement: Extended Data Figure 3-2 — Repetition suppression parameter estimates for the FFA, OFA, and primary visual cortex for analyses excluding targets that participants incorrectly classified (preregistered complementary analysis; N = 29). Upper panel, Repetition effects for all individuals. Lower panel, Effect size estimate and the bootstrapped 95% confidence intervals for the comparison between Black and White targets in each condition. As with the previous analysis, results replicate the main findings. A, FFA results. Activation in FFA was suppressed to nearly an equivalent degree for both Black and White repeated faces [repetition effect: F(1,28) = 180.11, MSE = 4.56, p < 0.001, η2p = 0.87; interaction effect: F(1,28) = 0.28, MSE = 1.60, p = 0.60, η2p = 0.01; equivalence test for the two repetition effects (mean difference: 0.25): p = 0.0598, equivalence interval (–1.05, 0.55)]. FFA also showed more release from suppression for White targets than for Black targets (F(1,28) = 6.94, MSE = 0.60, p = 0.01, η2p = 0.20; interaction between repeated and different faces: F(1,28) = 2.01, MSE = 0.94, p = 0.17, η2p = 0.07]. And again, similar to the main findings, we observed statistically equivalent levels of repetition suppression for different and repeated Black targets [equivalence test (mean difference: 0.47): p = 0.036, (–0.95, 0.02)]. B, The OFA demonstrated robust repetition suppression for repeated and different faces (F(1,28) = 233.52, MSE = 12.73, p < 0.001, η2p = 0.89 and F(1,28) = 267.83, MSE = 9.52, p < 0.001, η2p = 0.91 for repeated and different trials, respectively). The OFA showed some release from suppression for different faces (F(1,28) = 4.70, MSE = 3.46, p = 0.04, η2p = 0.14). As before, race did not interact with suppression for repeated or different faces (F(1,28) = 0.99, MSE = 7.52, p = 0.33, η2p = 0.03 and F(1,28) = 0.08, MSE = 4.51, p = 0.78, η2p = 0.003 for repeated and different targets, respectively). Thus, once again, the OFA was not sensitive to the group features of the di [file enu-eN-CFN-0431-19-s03.tif]

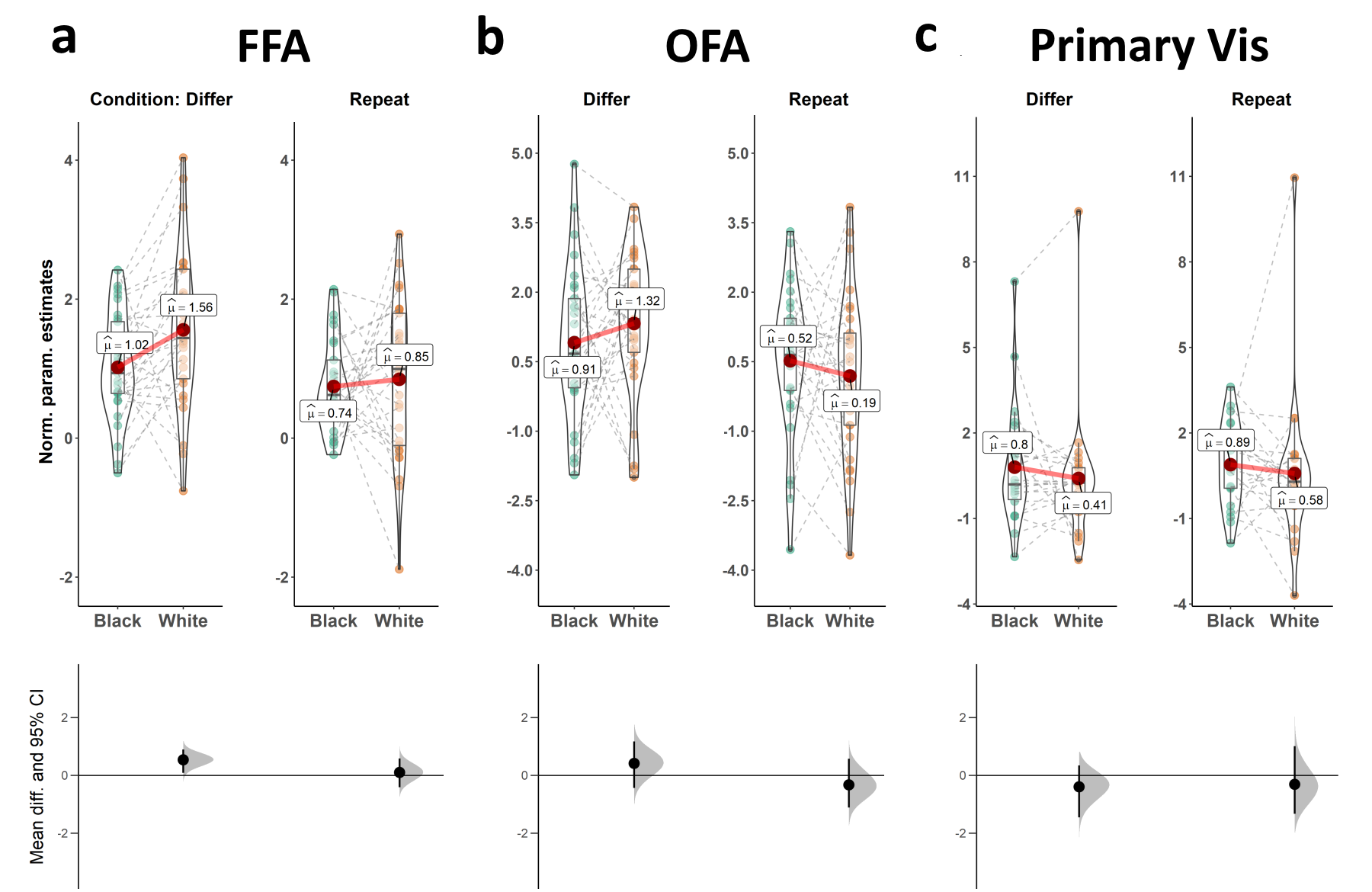

Supplement: Extended Data Figure 3-3 — Parameter estimates for the FFA, OFA, and primary visual cortex for the original preregistered analysis, in which we erroneously modelled single target trials with variable durations based on participants’ reaction time to the scrambled image that followed the single face (N = 29). Upper panel, Repetition effects for all individuals. Lower panel, Effect size estimate and the bootstrapped 95% confidence intervals for the comparison between Black and White targets in each condition. As in all previous analyses, we plot neural activity by subtracting the response to single faces (baseline) from the neural response to the different conditions (different, repeated), separately for each race (Black, White). Parameter estimates in this analysis are positive and no longer reflect suppression, as the baseline in this analysis reflects the neural activity in response to the scrambled image, rather than an actual face. This response was naturally weaker in face-sensitive regions. Nonetheless, overall pattern of results replicated the principal analysis. A, FFA results. Activation was different from baseline to an equivalent degree for Black and White repeated faces [repetition effect: F(1,28) = 51.79, MSE = 0.35, p < 0.001, η2p = 0.65; interaction effect: F(1,28) = 0.15, MSE = 0.50, p = 0.70, η2p = 0.005; equivalence test for the two repetition effects (mean difference: 0.10): p = 0.001, equivalence interval (–0.55, 0.34)]. FFA also showed more difference from baseline for White targets than for Black targets (F(1,28) = 6.74, MSE = 0.31, p = 0.01, η2p = 0.19; interaction between repeated and different faces: F(1,28) = 3.65, MSE = 0.38, p = 0.07, η2p = 0.12). And again, similar to the main findings, we observed statistically equivalent levels of difference from baseline for different and repeated Black targets [equivalence test (mean difference: 0.28): p < 0.001, (–0.55, –0.003)]. B, The OFA demonstrated difference from baseline only for different and not for repeated faces (F( [file enu-eN-CFN-0431-19-s04.tif]

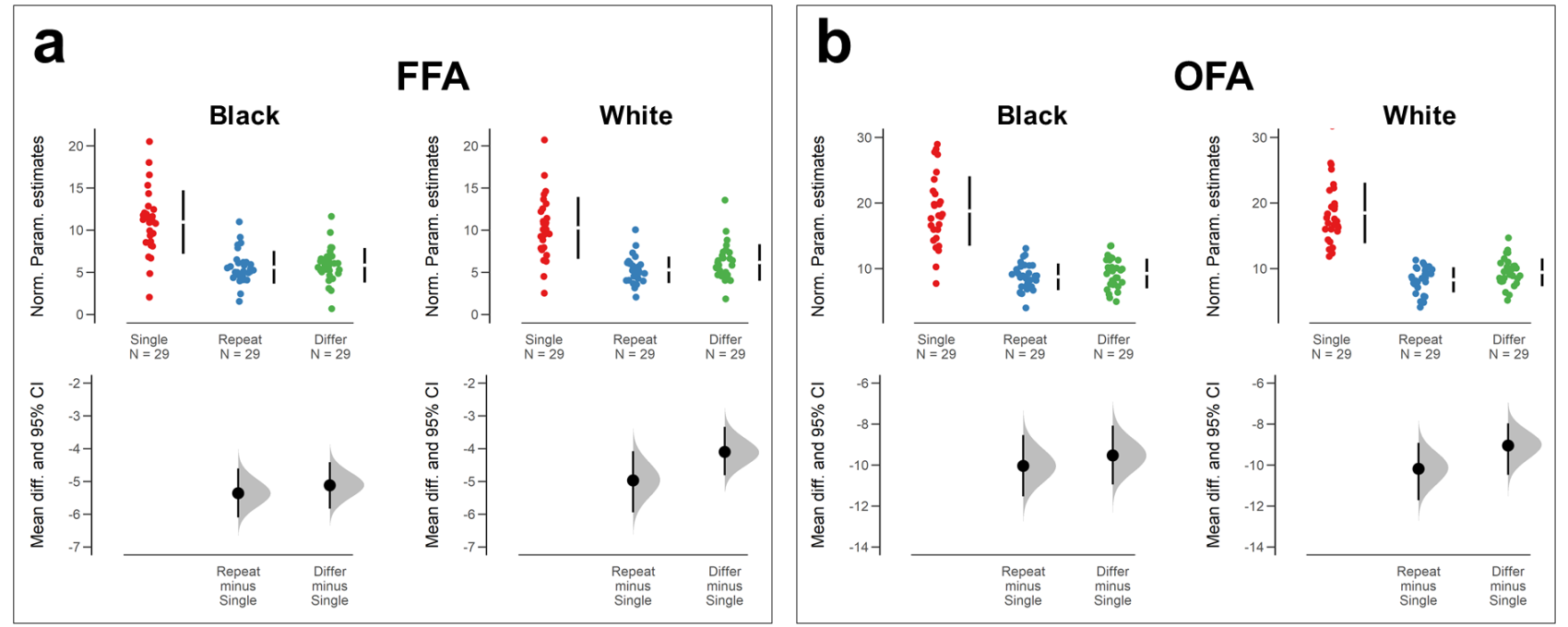

Supplement: Extended Data Figure 3-4 — Parameter estimates in the (A) FFA and (B) OFA for the three experimental conditions for Black and White male faces (without correcting for differences in baseline activity). Upper panels, Neural activity in response to the different conditions (single, different, repeated), separately for each race (Black, White). Individual dots represent neural activity for unique participants. Vertical lines (presented in parallel to the scatter plot) depict the mean and SD for each condition. Lower panel, Mean effect size (the repetition suppression effect) and the bootstrapped 95% confidence intervals for the comparison between type of repetition and single-face targets for each race. For statistical analyses, see Figure 3 and main text. Download Figure 3-4, TIF file. [file enu-eN-CFN-0431-19-s05.tif]

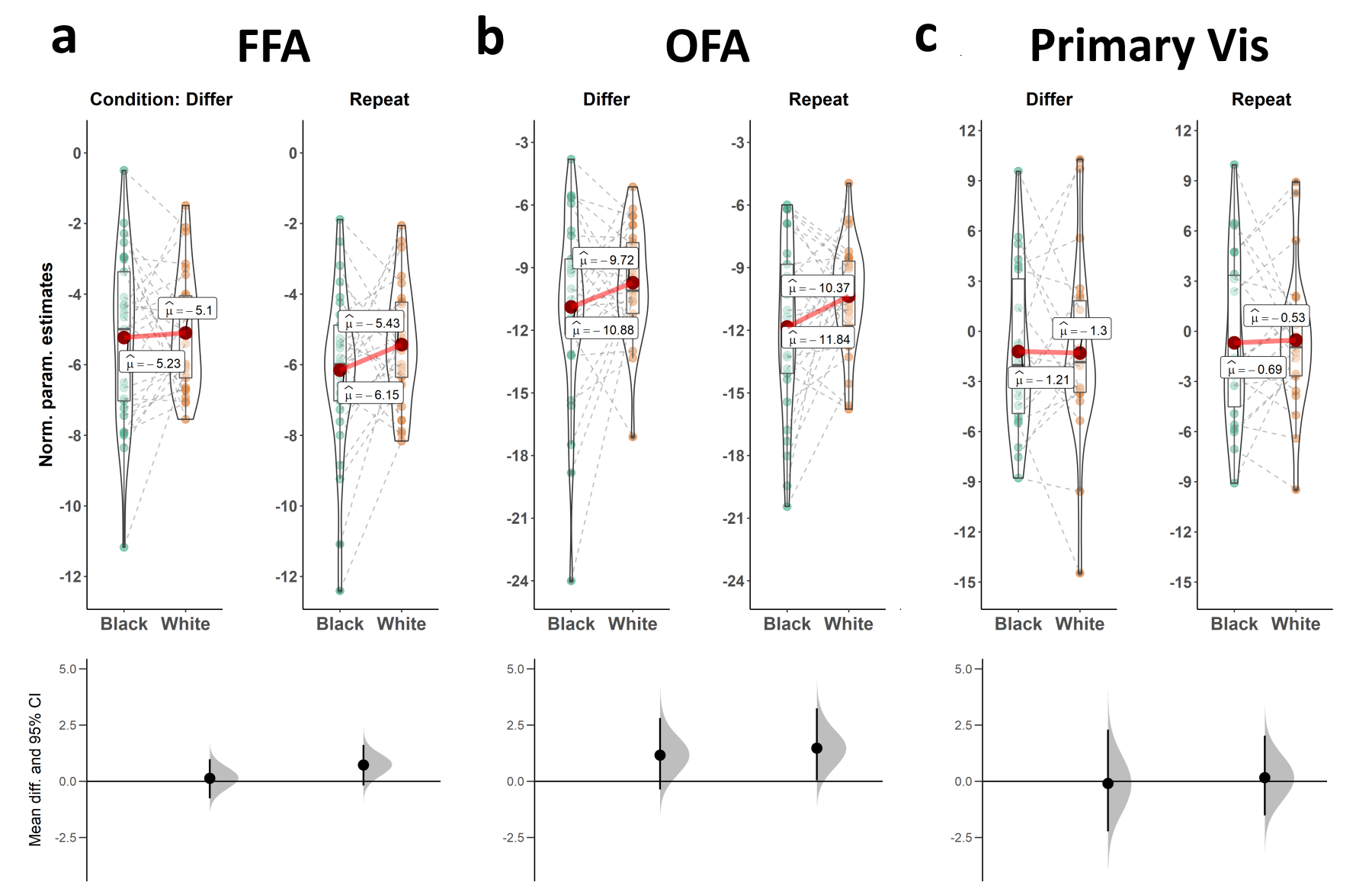

Supplement: Extended Data Figure 3-5 — Repetition suppression results for female targets for the FFA, OFA, and primary visual cortex using the analysis reported in the main manuscript (compare to results for male targets in Figs. 3B, 4). We subtracted neural activity in response to single faces (baseline) from the neural response to the different conditions (different, repeated), separately for each race (Black, White; upper panel). The lower panel depicts effect size estimate (the difference in suppression effect) and the bootstrapped 95% confidence intervals for the comparison between Black and White targets in each condition. A, The FFA demonstrated robust repetition suppression for repeated and different faces (F(1,28) = 392.66, MSE = 2.48, p < 0.001, η2p = 0.93 and F(1,28) = 290.91, MSE = 2.66, p < 0.001, η2p = 0.91 for repeated and different trials, respectively). The FFA also demonstrated release from suppression for different faces (F(1,28) = 14.66, MSE = 0.78, p < 0.001, η2p = 0.34), an effect that was qualified by an interaction with race (F(1,28) = 6.76, MSE = 0.38, p = 0.01, η2p = 0.19). However, the FFA did not show significant simple effects (i.e., differences in suppression) between Black and White female targets (F(1,28) = 2.41, MSE = 1.58, p = 0.13, η2p = 0.08 and F(1,28) = 0.08, MSE = 1.51, p = 0.77, η2p = 0.003 for repeated and different faces, respectively). As we did not have an a priori hypothesis for female targets, we cannot offer a reliable interpretation of these results. B, Much like for male targets, the OFA demonstrated robust repetition suppression for repeated and different faces (F(1,28) = 517.23, MSE = 6.92, p < 0.001, η2p = 0.95 and F(1,28) = 371.93, MSE = 8.28, p < 0.001, η2p = 0.93 for repeated and different trials, respectively). The OFA showed some release from suppression for different faces (F(1,28) = 6.70, MSE = 2.79, p = 0.02, η2p = 0.19), but no indication of an effect of race on any of these results (F(1,28) = 3.25, MSE = 4.84, p = 0.08, η2p = 0.10 and F(1,28) [file enu-eN-CFN-0431-19-s06.tif]
