# Supplementary material for: Human Face-Selective Cortex Does Not Distinguish between Members of a Racial Outgroup
Source: eNeuro. 2020 May 29;7(3):ENEURO.0431-19.2020. doi: 10.1523/ENEURO.0431-19.2020 (PMC7266143; doi:10.1523/ENEURO.0431-19.2020)
Supplement: Extended Data Figure 3-6 — Results from the main functional ROI analysis [group-constrained subject-specific (GcSS); see Materials and Methods]. For each ROI, the GcSS algorithm defines a parcel to intersect with activation for each individual participant, thus reliably identifying the same functional ROIs in all participants. We conducted the GcSS analyses with the contrast of faces > other categories in independent localizer scans. A priori ROIs are presented in italics. Download Figure 3-6, DOCX file. [file enu-eN-CFN-0431-19-s07.docx]

| ***Region Name*** | ***% Participants in parcel*** | ***# Voxels (parcel)*** | ***Average # voxels per individual participants*** | ***Location of peak overlap (MNI)*** | | |
| --- | --- | --- | --- | --- | --- | --- |
|  |  |  |  | ***X*** | ***Y*** | ***Z*** |
| *R occipital face area (rOFA)* | 100% | 1819 | 510 | 41 | -79 | -6 |
| *R fusiform gyrus (rFFG)* | 100% | 1271 | 362 | 40 | -51 | -18 |
| R superior temporal sulcus | 100% | 1888 | 492 | 56 | -39 | 9 |
| L posterior fusiform gyrus (lpFFG) | 100% | 490 | 119 | -40 | -60 | -18 |
| L anterior fusiform gyrus (laFFG) | 97% | 315 | 74 | -40 | -46 | -20 |
| L occipital face area (lOFA) | 100% | 1191 | 260 | -39 | -81 | -8 |
| Early visual cortex | 76% | 1030 | 224 | 2 | -86 | 18 |
| R posterior superior temporal sulcus | 97% | 764 | 169 | 51 | -59 | 12 |
| Cuneus | 72% | 527 | 104 | 5 | -83 | 35 |
| L posterior superior temporal sulcus (lpSTS) | 100% | 989 | 176 | -53 | -59 | 14 |
| R motor cortex | 93% | 477 | 86 | 46 | 4 | 50 |
| L superior temporal sulcus | 90% | 234 | 44 | -59 | -41 | 8 |
| R inferior frontal gyrus | 69% | 424 | 75 | 46 | 20 | 24 |
| R anterior superior temporal sulcus | 76% | 390 | 66 | 55 | -13 | -10 |
| Dorsal medial prefrontal cortex | 66% | 232 | 37 | 4 | 58 | 19 |
| R anterior inferior frontal gyrus | 62% | 266 | 41 | 52 | 34 | 1 |
| Retrosplenial cortex | 59% | 230 | 35 | 2 | -66 | 36 |
| Retrosplenial cortex | 45% | 169 | 26 | 2 | -54 | 36 |
| R motor cortex | 48% | 98 | 16 | 7 | 11 | 72 |
| Medial prefrontal cortex | 59% | 96 | 15 | 6 | 60 | 9 |
| R anterior superior temporal sulcus | 66% | 95 | 15 | 52 | 8 | -21 |
| R Amygdala | 45% | 19 | 4 | 19 | -4 | -16 |
| R Anterior temporal lobe | 41% | 74 | 11 | 47 | -18 | -30 |
